# Metabolomics of Therapy Response in Preclinical Glioblastoma: A Multi-Slice MRSI-Based Volumetric Analysis for Noninvasive Assessment of Temozolomide Treatment

**DOI:** 10.3390/metabo7020020

**Published:** 2017-05-18

**Authors:** Nuria Arias-Ramos, Laura Ferrer-Font, Silvia Lope-Piedrafita, Victor Mocioiu, Margarida Julià-Sapé, Martí Pumarola, Carles Arús, Ana Paula Candiota

**Affiliations:** 1 Departament de Bioquímica i Biologia Molecular, Unitat de Bioquímica de Biociències, Edifici Cs, Universitat Autònoma de Barcelona, 08193 Cerdanyola del Vallès, Spain; Nuria.Arias@uab.cat (N.A.-R.); Laura.Ferrer@uab.cat (L.F.-F.); Carles.Arus@uab.cat (C.A.); 2 Centro de Investigación Biomédica en Red en Bioingeniería, Biomateriales y Nanomedicina (CIBER-BBN), 28029 Madrid, Spain; Margarita.Julia@uab.cat; 3 Institut de Biotecnologia i de Biomedicina (IBB), Universitat Autònoma de Barcelona, 08193 Cerdanyola del Vallès, Spain; victormocioiu@gmail.com; 4 Servei de Ressonància Magnètica Nuclear, Edifici C, Universitat Autònoma de Barcelona, 08193 Cerdanyola del Vallès, Spain; Silvia.Lope@uab.cat; 5 Departament de Medicina i Cirurgia Animals, Facultat de Veterinària, Edifici V, Universitat Autònoma de Barcelona, 08193 Cerdanyola del Vallès, Spain; Marti.pumarola@uab.cat

**Keywords:** glioma, GL261, orthotopic tumors, therapy response, TMZ, immune response, nosological images

## Abstract

Glioblastoma (GBM) is the most common aggressive primary brain tumor in adults, with a short survival time even after aggressive therapy. Non-invasive surrogate biomarkers of therapy response may be relevant for improving patient survival. Previous work produced such biomarkers in preclinical GBM using semi-supervised source extraction and single-slice Magnetic Resonance Spectroscopic Imaging (MRSI). Nevertheless, GBMs are heterogeneous and single-slice studies could prevent obtaining relevant information. The purpose of this work was to evaluate whether a multi-slice MRSI approach, acquiring consecutive grids across the tumor, is feasible for preclinical models and may produce additional insight into therapy response. Nosological images were analyzed pixel-by-pixel and a relative responding volume, the *Tumor Responding Index* (*TRI*), was defined to quantify response. Heterogeneous response levels were observed and treated animals were ascribed to three arbitrary predefined groups: high response (HR, *n* = 2), *TRI* = 68.2 ± 2.8%, intermediate response (IR, *n* = 6), *TRI* = 41.1 ± 4.2% and low response (LR, *n* = 2), *TRI* = 13.4 ± 14.3%, producing therapy response categorization which had not been fully registered in single-slice studies. Results agreed with the multi-slice approach being feasible and producing an inverse correlation between *TRI* and Ki67 immunostaining. Additionally, ca. 7-day oscillations of *TRI* were observed, suggesting that host immune system activation in response to treatment could contribute to the responding patterns detected.

## 1. Introduction

Glioblastoma (GBM) is the most common and aggressive glial primary tumor with a survival average of 14–15 months, even after application of standard treatment [1]. Temozolomide (TMZ) plus radiotherapy is the regular therapeutic choice for such treatment [2] and produces the best survival rates.

Magnetic resonance techniques are the most suitable approaches to perform diagnosis and therapy response follow-up in brain tumors as GBM. These techniques could provide anatomical information (MRI, magnetic resonance imaging) or information of the metabolomic profile (MRS, magnetic resonance spectroscopy). Still, magnetic resonance spectroscopic imaging could provide both, metabolomic information superimposed to anatomical information. Namely, the difference between MRS and MRSI is that MRS consists in acquiring single spectrum from a certain volume, whereas in MRSI multiple signals from a grid of voxels are acquired, allowing to gather metabolomic information from different regions of the studied tissue [3].

Radiological and clinical guidelines are used to evaluate GBM response to therapy, particularly through the application of the response assessment in neuro-oncology (RANO) criteria [4] and Response Evaluation Criteria in Solid Tumors (RECIST) criteria [5]. Nevertheless, magnetic resonance imaging (MRI) information is not always precise enough for this due to pseudoresponse and pseudoprogression appearance in some cases [6]. In this sense, magnetic resonance spectroscopy (MRS) and magnetic resonance spectroscopic imaging (MRSI) might be a useful supplementary tool for this purpose [7], as MRS/MRSI are also used for monitoring the molecular properties and metabolic heterogeneity of brain tumors [8,9]. Furthermore, the rich information contained in MRS/MRSI signals makes them ideally suited to the application of statistical pattern recognition (PR) techniques [10], which are used nowadays to perform automatic categorization of individual MRSI data obtained from different types of tissue. This approach can be applied to MRSI data from human brain tumors [11] and preclinical animal models [12]. PR techniques have also been proven useful to detect and characterize tumor response to therapy [13] with generation of nosological images of therapy response. Briefly, nosological images are those in which each pixel is colored according to a class (e.g., tumor and normal tissue). For MRSI, each pixel corresponds to an individual spectrum, and, in our study, pixel classification is performed by mathematical PR.

Additionally, Ki67 staining for proliferation rate has been reported as a good response biomarker for preclinical [14] and clinical GBM, also related to patient survival and time to recurrence [15]. However, Ki67 studies require invasive procedures (biopsy sampling), and thus the development of non-invasive techniques that would correlate with this biomarker would be highly desirable.

The preclinical glioblastoma model of GL261 cells growing into C57BL/6 mice has been used for more than 20 years in different therapy evaluation approaches [16,17,18]. In addition, work from our group on TMZ-treated C57BL/6 mice harboring orthotopic GL261 GBM has demonstrated an increase in survival rate (33.9 ± 11.7 days, *n* = 38), compared to control GL261 GBM mice with no treatment (21.50 ± 3.7 days, *n* = 61), from [13,18] and additional unpublished data from our group.

In previous studies from our group, we could demonstrate that there was a significant correlation when comparing the MRSI-based nosological maps with the proliferation rate (Ki67) of the tumors (higher proliferation rate meaning lack of response or relapse in case of treated tumors) [13]. However, only the central part of the tumor was analyzed (single MRSI slice). Thus, we thought that it would be of interest to evaluate the tumors using a multi-slice approach, as it has been demonstrated that GBMs display intratumoral heterogeneity [19,20] that may influence tumor aggressiveness or response to chemotherapies such as TMZ [21]. Accordingly, in this work, we have set-up a multi-slice protocol for MRSI evaluation, and performed as well the correlation of nosological images with the proliferation rate of the tumors through Ki67 immunostaining. Our goal was to determine if substantial differences could be proven to exist in the different zones of the tumor undergoing classical TMZ administration protocol and sampled by nosological imaging, as observed in previous studies from our group [13,18].

## 2. Results

Two groups of treated mice were used in this work (please refer to Materials and Methods Section 4.2 and Section 4.3 for further detail on groups). All spectra from MRSI matrices were aligned before performing PR analysis (see Section 4.4.2 of Materials and Methods for additional details on preprocessing).

### 2.1. Group A: Cases Starting Therapy at Day 11 p.i.: Inclusion into Different Groups of Response Level

Twelve animals were chosen for longitudinal multi-slice MRSI studies (see Materials and Methods Section): six mice (C971, C974, C975, C1022, C1023 and C1026) were classified as intermediate response (IR) cases and two mice (C979 and C981) were classified as low response (LR) cases. All but one case (C974) were euthanized at the time point for which criteria for inclusion into IR or LR groups were accomplished. Regarding case C974 (IR), it was maintained alive until the endpoint to follow-up the possible *TRI* changes along time. No high response (HR) cases were found in this first part of the experiment (the maximum calculated *TRI* was of 46.5%, see also Section 2.2). In addition, four control cases (C1109, C1110, C1111 and C1112) were also analyzed with multi-slice MRSI studies, although only one measurement was carried out at Days 11 p.i., 13 p.i., 16 p.i. and 13 p.i., respectively, and they were euthanized when tumor volumes were about 70.2 ± 18.7 mm^3^.

It is worth mentioning that in addition to the four control cases explored in this work, 28 additional cases from previous work from our group were added to confirm consistent evolution of the pattern by MRI follow-up, reaching a total of *n* = 32 control cases (Figure S1).

Table S1 shows relevant parameters for these cases: tumor volume pre-treatment, tumor volume at euthanasia, RECIST criteria classification of response to therapy and *TRI* criteria classification of response to therapy).

In addition, significant differences (*p* < 0.05) were observed between body weight evolution between all treated groups (Figure S2).

#### 2.1.1. *TRI* and Nosological Images Evolution vs. Tumor Volume Evolution

**Figure 1:**
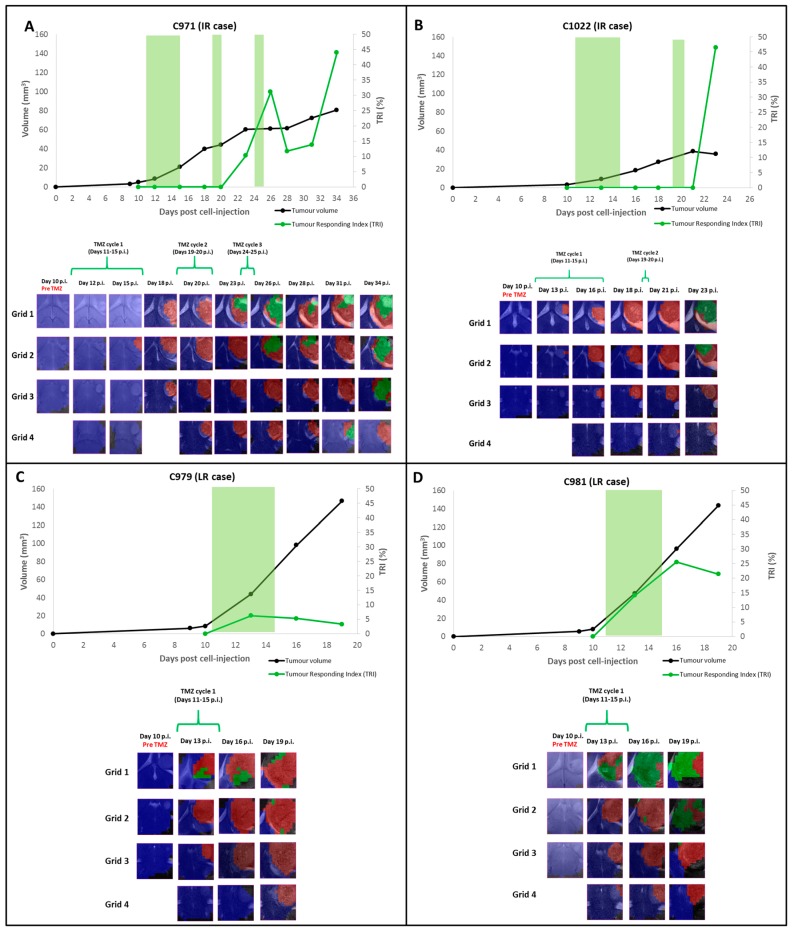
Graphical representation of the tumor volume evolution (in mm^3^, black line, left axis), and the percentage of responding “green” pixels obtained after application of source analysis to MRSI data acquired in the multi-slice set as in [13] (in percent, green line, right axis). The green shaded columns indicate TMZ administration periods. For chosen time points, the evolution of the nosological images obtained with the semisupervised source extraction system is shown in four columns of color coded grids, superimposed to the T_2w_-MRI for each slice (color coding as follows: blue pixels: normal parenchyma, red pixels: non responding, green pixels: responding). Green brackets indicate TMZ administration periods. (**A**) Corresponds to the IR case C971, green pixels were observed first at Day 23 p.i., increasing until 32.2% at Day 26 p.i., when tumor growth arrest was observed. Then, *TRI* decreased: 11.7% at Day 28 p.i. and 13.9% at Day 31 p.i., followed by a new increase up to 44.1% at Day 34 p.i. (**B**) Corresponds to the IR case C1022, only red tumor pixels were observed in MRSI measurements until Day 23 p.i., tumor volume decrease was observed and green pixels were observed in Grids 1 and 2 (*TRI* = 46.5%). (**C**) Corresponds to the LR case C979, green pixels were observed only in Grid 1 at Days 13 p.i., 16 p.i. and 19 p.i. (*TRI* = 6.3%, 5.2%, 3.3%, respectively), tumor growth arrest was not observed. (**D**) Corresponds to the LR case C981, green pixels were observed only in Grid 1 at Days 13 and 16 p.i. At Day 23 p.i., green pixels were observed in Grids 1 and 2 (*TRI* = 21.5%).

**Figure 2:**
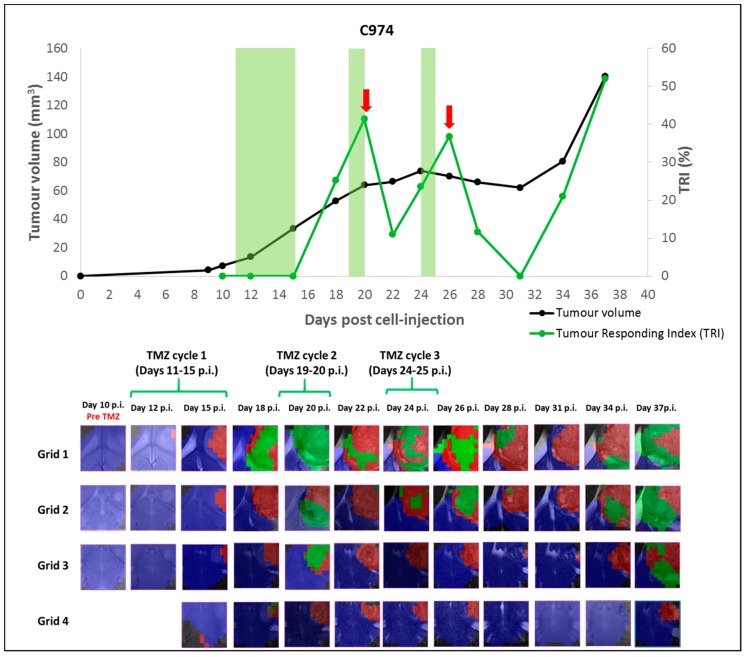
Graphical representation of the tumor volume evolution (in mm^3^, black line, left axis), and the percentage of responding “green” pixels obtained after source analysis of MRSI data acquired in the multi-slice set as in [13] (in percent, green line, right axis) for case C974 (IR case). The green shaded columns indicate TMZ administration periods. For chosen time points, the evolution of the nosological images obtained with the semisupervised source extraction system is shown in four columns of color coded grids, superimposed to the T_2w_-MRI for each slice. Green brackets indicate TMZ administration periods. A cyclical pattern of response was observed, marked by the red arrows in the graphical representation.

*IR cases:* the relationship between *TRI* and tumor volume evolution of IR cases is shown in Figure 1 and Figure 2. Euthanasia was performed when *TRI* values met the inclusion criteria for IR class, namely: *TRI* ranging between 35–65% and tumor volume meeting criteria for “stable disease” according to RECIST (see Material and Methods, Section 4.5 for *TRI* calculation). These criteria were met in five out of six IR cases at the euthanization time. The mean *TRI* value of these five cases (C971, C975, C1022, C1023 and C1026) was 44.1 ± 4.2%. The remaining case (C974) met IR conditions during part of the evolution period, and was not euthanized but followed-up until endpoint, and will be described in a separate section. In IR cases, *TRI* values increased after the second therapy cycle, coinciding with tumor growth arrest (stable disease stage by RECIST criteria). The maximum variation in tumor volume in the stable disease period of IR cases was of 17.6%. Figure 1 shows two chosen examples of IR cases: C971 and C1022 (Figure 1A,B, respectively). In case C1022, *TRI* increase (46.5%) was found four days after the second therapy cycle, during tumor growth arrest. On the other hand, in case C971 (Figure 1A), we can observe that *TRI* also increased its value during the “stable disease” stage (31.2% at Day 26 p.i., six days after the second therapy cycle), but it did not follow a continuous increasing trend during the whole tumor development. In fact, it experienced a decrease during Days 28–31 p.i. (*TRI* 11.7% and 13.9%, respectively) and a further increase (44.1% at Day 34 p.i., nine days after the third therapy cycle), when the mouse was euthanized for histopathological validation. The interval between the two *TRI* peak values was of eight days.

Other examples of IR cases can be found in the Supplementary Material. For example, in case C975 (Figure S3A), *TRI* increase was first observed at Day 20 p.i., just after the second therapy cycle, with further increase at 26 p.i. (*TRI* = 40.3%), six days after the second therapy cycle. In cases C1023 and C1026 (Figure S3B,C), *TRI* start increasing at Day 18 p.i., after the first therapy cycle with further increases (35.8% and 38.9%, respectively); at Day 23 p.i., four days after the second therapy cycle; and eight days after the first cycle. These *TRI* increases were in the same time frame as in case C1022.

Nosological images acquired for IR cases are also shown in the aforementioned figures. In general, green responding pixels started to be observed in Grid 1 in all IR cases and then “spread” to the remaining grids (with a predominance in the upper grids: Grid 1 and Grid 2). Accordingly, there was a variation in the response level in different grids taking into account the detection of green pixels, with higher *TRI* values being found in the upper grids.

*LR cases:* (C979 and C981) did not respond to therapy with TMZ and they had to be euthanized due to welfare parameters (see Table S2) at Day 19 p.i. (their body weight loss was higher than 20% compared to the day pre cell injection, C979: 51.55% and C981: 54.22%, before starting the second therapy cycle). The mean *TRI* value of these two cases was 13.4 ± 14.3%. In Figure 1C,D, the relationship between tumor volume and *TRI* evolution as well as nosological images for cases C979 and C981 are shown.

Very few green responding pixels were observed in the nosological images of case C979 and only in the first grid (*TRI* = 3.3% at the euthanasia day). In case C981, green pixels were observed first in Grid 1 and at the euthanasia day in Grids 1 and 2, but *TRI* value was higher than case C979, reaching a value of 21.5%.

#### 2.1.2. Case C974: Tracking the Evolution of an IR Case along Time

This was a case for which euthanasia was not performed at an intermediate tumor evolution point according to *TRI* for further histopathology analysis and validation. Indeed, the tumor was allowed to evolve until endpoint in order to assess *TRI* changes all along the tumor growth curve. The tumor volume evolution and its relationship with *TRI*, as well as the corresponding nosological images obtained, are shown in Figure 2. The *TRI* started increasing at Day 18 p.i., three days after finishing the first therapy cycle, reaching a value of 41.4% at Day 20 p.i. (just after finishing the second cycle). At this particular moment, although *TRI* values would meet criteria for classification as IR, the 21% increase in tumor volume would not agree with “stable disease” according to RECIST criteria. Then, from Day 20 to Day 31 p.i., volume growth arrest was observed (“stable disease” stage by RECIST criteria), with maximum volume variation of 11.2% regarding previous measurements. During this period of growth arrest, a cyclical pattern in *TRI* values was observed, as follows: *TRI* value decreased to 11% at Day 22 p.i. (between cycles 2 and 3), then increased again reaching a value of 36.8% at Day 26 p.i. (one day after finishing the third therapy cycle), moment at which criteria for IR would be accomplished for this case. Further *TRI* decreases to 11.7% and 0% were seen at Days 28 and 31 p.i. respectively.

Finally, progressive disease stage by RECIST criteria was observed between Days 34 p.i. and 37 p.i. (the last measurement, in which tumor volume increased 29.9% and 73.9% regarding the previous measurements), and *TRI* during this period also increased to 21.1% and 52.1% values respectively. This mouse died during MRI acquisition at Day 40 p.i. During this period, body weight loss (compared to the body weight observed at the day of cell injection) was of 7.3% at Day 34 p.i. and 33% at Day 40 p.i., in agreement with frank disease progression.

In the nosological images, green pixels were observed first in the upper grid, as in the other IR cases. It is worth noting that the *TRI* high peaks were observed 5–6 days after the first and second therapy cycle.

**Figure 3:**
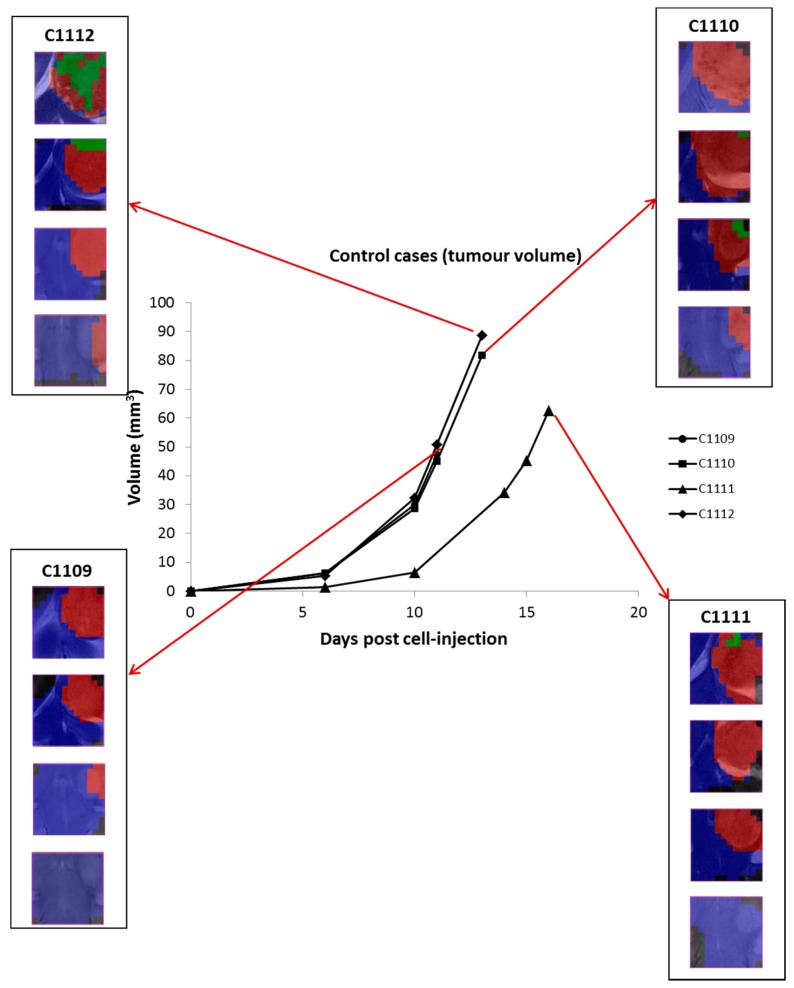
Tumor volume evolution (in mm^3^, black line) of untreated, control cases (C1109, C1110, C1111 and C1112). Only one multi-slice MRSI measurement was carried out in these cases. Red arrows point to the nosological images obtained with the source analysis system (four columns of color coded grids, superimposed to the T_2w_-MRI for each slice). *TRI* values obtained for each case were: 0% (C1109), 3.5% (C1110), 1.9% (C1111) and 19.3% (C1112).

*Control cases:* Tumor volumes of four control mice (C1109, C1110, C1111 and C1112) are shown in Figure 3. These mice were studied with multi-slice MRSI acquisition (a single measurement, carried out at the same day these animals were euthanized), and *TRI* was also calculated (average *TRI* value for all cases was of 6.2 ± 2.8%). In case C1109, no green pixels were observed. However, green responding pixels were also found in the other control cases: in case C1110, a total of six green pixels were observed after analyzing all grids (*TRI* = 3.5%); in case C1111 only three pixels were observed (*TRI* = 1.9%); and in case C1112, green pixels were observed in Grids 1 and 2 (*TRI* = 19.3%).

### 2.2. Group B: Therapy Response in Cases Starting Therapy with Tumor Volume 3–5 mm^3^: Finding High Response (HR) Cases

As no HR cases were found in the first part of the experiment, the objective of this second part of the study was to introduce modifications in the therapy protocol in order to increase the probability of finding HR cases. For this, therapy administration started when tumor size was between 2.6–5.2 mm^3^, disregarding the day p.i. of the tumors.

**Figure 4:**
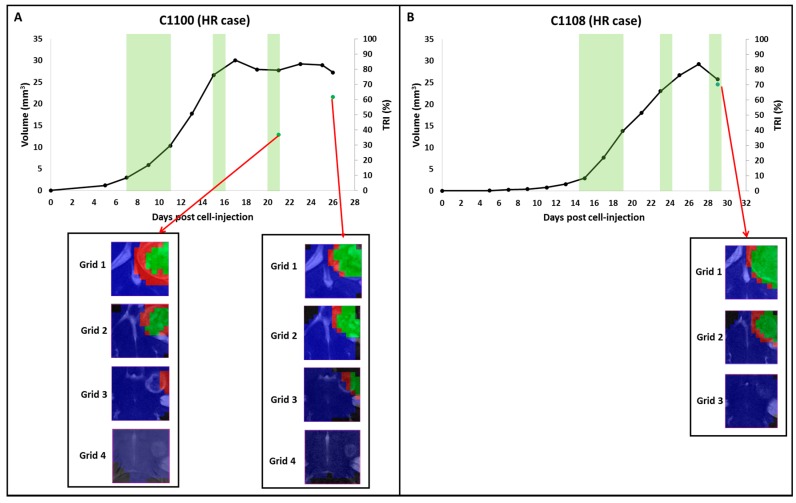
Graphical representation of the tumor volume evolution (in mm^3^, black line, left axis), and the percentage of responding “green” pixels obtained after source analysis of MRSI data acquired in the multi-slice set as in [13] (in percent, green dots, right axis) of HR cases. The green shaded columns indicate TMZ administration periods. For chosen time points, the evolution of the nosological images obtained with the semisupervised source extraction system is shown, superimposed to the T_2w_-MRI for each slice. The number of grids (3 or 4) depended of the coverage of the tumor: if tumor was totally covered with three grids, no additional grids were acquired. (**A**) Corresponds to the case C1100: green pixels were observed in Grids 1 and 2 with a *TRI* = 36.8% at Day 21 p.i. during tumor growth arrest. Then at Day 26 p.i., tumor volume decreased and a *TRI* of 61.8% was observed. (**B**) Corresponds to the case C1108: green pixels were observed in Grids 1 and 2 with a *TRI* = 70.3% when tumor volume decrease was observed.

From 15 mice which were followed up by MRI, 11 of them started therapy administration at Day 7 p.i., whereas the other four mice started therapy at Days 9, 11, 13 and 15 p.i., respectively. The mean tumor size of these cases at the starting therapy day was of 3.8 ± 0.8% mm^3^. From these 15 mice, only two (C1100 and C1108) presented measurable decrease in tumor volume (5.9% and 12.0%, respectively) compared to the previous day of measurement, which was a criterion set by us to start the multi-slice MRSI acquisitions. During follow up, these two cases met the requirements for being classified as HR cases (*TRI* > 65% and volume decrease respect the previous day of measurement). *TRI* and other parameters of these cases are shown in Section 2.4. The mean *TRI* value found in HR cases was 68.2 ± 2.8%. Evolution of tumor volume and nosological images of C1100 and C1108 are shown in Figure 4.

For case C1100, therapy administration started at Day 7 p.i., when tumor volume was 3.0 mm^3^. This mouse was explored by MRI until Day 17 p.i. (six days after finishing the first therapy cycle), when a 7.1% decrease in volume was observed compared to the previous measurement. From Day 17 p.i. to Day 21 p.i., tumor growth arrest was observed in MRI measurement. At Day 21 p.i., a 0.6% of volume decrease was detected and a multi-slice MRSI measurement was performed and the calculated *TRI* was 36.8%. The next measurement took place at Day 26 p.i. when a *TRI* of 61.6% was detected and a most clear decrease in volume was observed with respect to the previous measurement (5.9%). The mouse was then euthanized for histopathological validation. In the nosological images (Figure 4A), at the first day of MRSI acquisition, green pixels were observed in the first two upper grids and in the last day of measurement, green pixels were observed in Grids 1, 2 and 3 (in the fourth grid, the tumor could not be properly segmented with our analysis, probably due to the small tumor area found in this grid, composed mostly by normal/peritumoral tissue).

Regarding case C1108, therapy administration started at Day 15 p.i., when tumor volume was 2.9 mm^3^. This mouse was explored by MRI until Day 29 p.i. (five days after finishing the second therapy cycle, when a 12% decrease in volume was observed compared to the previous measurement). The value found for *TRI* that day was 70.3% and the mouse was euthanized for histopathological validation. In nosological images (Figure 4B), green pixels were observed in Grid 1 and Grid 2 (in Grid 3, the tumor could not be segmented, as previously mentioned for case C1100).

Body weight loss of both cases was less than 20% the day euthanasia was performed (C1100: 9% and C1108: 11.5%). Accordingly, significant differences (*p* < 0.05) were observed in body weight evolution between HR and IR cases, between HR and LR cases and between IR and LR cases (see also Figure S2).

### 2.3. Metabolic Pattern Contributing to Responding and Non-Responding (Red and Green) Areas Detected in MRSI Studies of the Investigated Mice

**Figure 5:**
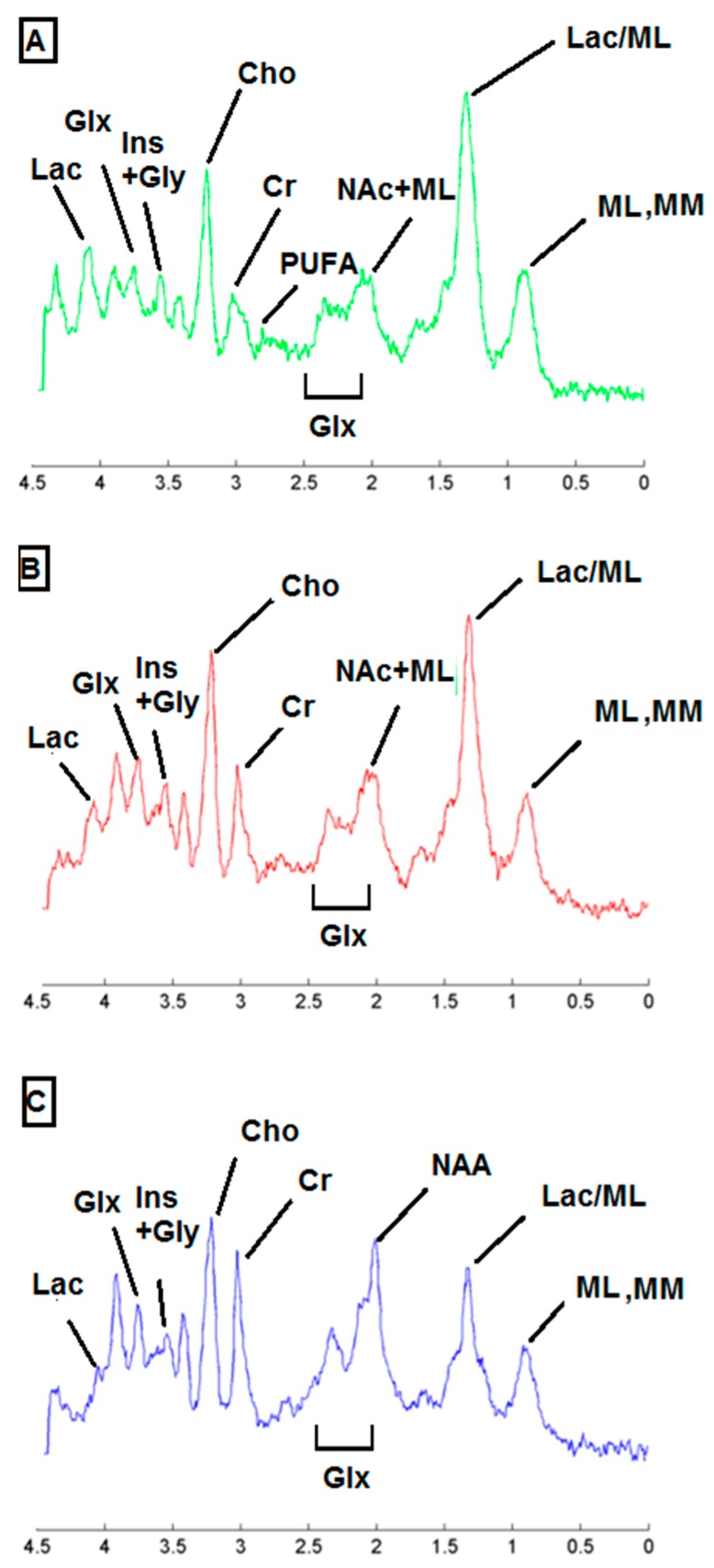
Example of mean spectra extracted from the MRSI grid of a treated case (HR case C971: Grid 1, Day 34 pi): (**A**) mean spectra of responding area (*n* = 26); (**B**) mean spectra of non-responding area (*n* = 35); and (**C**) mean spectra of normal brain parenchyma area (*n* = 39). Some of the main metabolites contributing to different patterns of response to therapy are shown: mobile lipids 0.9 + macromolecules (ML, MM, 0.9 ppm), mobile lipids 1.3 + lactate (ML/Lac, 1.3 ppm), *N*-acetyl-aspartate and *N*-acetyl group containing compounds (NAA and NAc, 2.02 ppm) (see also [31]), glutamate + glutamine (Glx, 2.1–2.4 ppm) polyunsaturated fatty acids in mobile lipids (PUFA, 2.8 ppm), total creatine (Cre, 3.03 ppm), choline-containing compounds (Cho, 3.21 ppm), myo-inositol + glycine (Ins + Gly, 3.55 ppm), glutamine + glutamate (Glx, 3.8 ppm, which is also partially contributed by alanine), and lactate (Lac, 1.3 and 4.1 ppm).

Different metabolites have been described to contribute to the spectral pattern of preclinical glioblastoma [13,18,22,23,24,25,26,27,28,29] and some of them are listed in Table 1, being also observed in cases analyzed in this study. Figure 5 shows examples of average spectra extracted from blue, red and green zones of a HR case (C971), whereas Figure S4 show examples of average spectra from red and green zones in comparison with the sources extracted from treated and untreated cases, reported in [13]. It is worth mentioning that the changes observed in the whole metabolic pattern, rather than isolated changes in few metabolites, is what allows the semi-supervised source extraction system to classify MRSI voxels in different classes (normal brain, responding tumor and non-responding tumor) [13].

The main differences between normal brain parenchyma and tumor areas (blue vs. green/red areas) were: higher choline/creatine (Cho, 3.21/Cre, 3.03 ppm) ratios, higher mobile lipids (ML, 0.9 and 1.3 ppm) and lactate (Lac, 1.3 ppm and 4.1 ppm) peak intensities in tumor areas, as well as lower *N*-acetyl-aspartate (NAA/NAc, 2.02 ppm) signal, already described by us in [18]. The zones classified as “normal brain parenchyma” by the source extraction approach presented the expected features, such as lower ML and ML/Lac signals in comparison with tumors, and a ca. 1:1 ratio for Cho and Cre (see [26] for more examples of control spectra in mice brain).

Although the responding and non-responding spectra (which are present in green and red areas respectively) could seem quite similar, relevant differences between them are sampled by the source analysis especially related to polyunsaturated fatty acids in ML (PUFA, 2.8 ppm), as already described by [18,30]. Other minor contributions are Lac (4.1 ppm), glutamine plus glutamate and alanine (Glx + Ala, 3.8 ppm) and myo-inositol and glycine (Ins + Gly, both seen at 3.55 ppm).

### 2.4. Histopathology Validation

Cases used for histopathological validation were, according to *TRI* response: two HR cases (C1100 and C1108), three IR cases (C971, C1022 and C1026), one LR case (C979) and two Control cases (C1110 and C1111).

**Figure 6:**
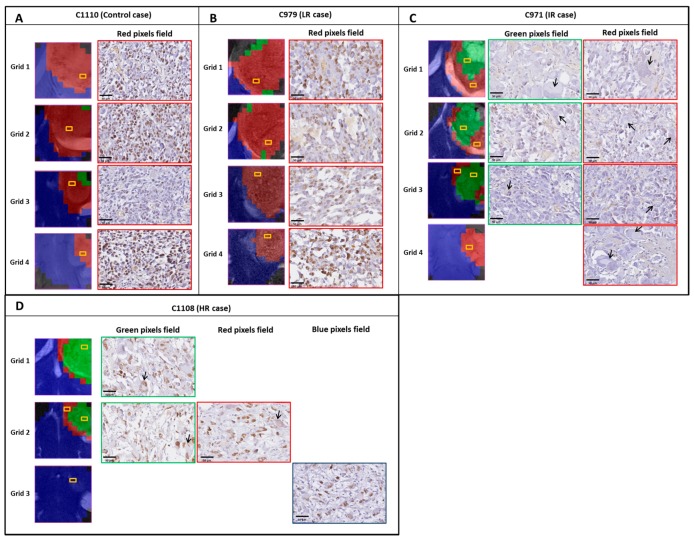
Ki67 immunostainings (40× magnification, the scale bar corresponds to 50 µm) in histological areas corresponding to red or green regions in nosological images, from different grids of chosen cases. The yellow rectangles in the nosological image identify the approximate origin of the histopathological preparations shown. The nuclei of Ki67 positive cells are stained in brown. (**A**) Control case C1110, with a global Ki67 of 63.9 ± 13.0% and a highly dense cellular population showing a typical tumoral morphology with small to medium-sized polygonal or irregular cells with rounded nuclei and scanty cytoplasm without any giant cells visible. (**B**) LR case, C979: in this case, Ki67 positive cells were also observed (64.8 ± 7.3%) but with less cellular density due to presence of large acellular spaces in comparison with the control case. (**C**) IR case C971 presented lower Ki67 immunostaining (22.0 ± 17.2%) than control and LR cases, with presence of giant cells with several nuclei (black arrows), and also acellular spaces. No significant differences were observed for Ki67 between red and green areas from nosological images of this case, although the trend to higher Ki67 in red areas is maintained (Ki67 for red areas 25.7 ± 17.0% and Ki67 for green areas 17.2 ± 16.8%). (**D**) HR case C1108, with an average Ki67 of 79.3 ± 10.1%, although it is worth noting that HR cases present lower number of total tumoral cells per field in comparison with IR cases (57.1 ± 16.9 and 86.7 ± 63.9 cells/field respectively), which could have an influence in the final result of Ki67% calculation. Still, acellular areas and giant cells (black arrows) were also observed. See Discussion Section for a possible explanation with respect to apparently high Ki67 values in green nosological image regions.

Haematoxylin/Eosin morphology analysis: different cell morphology (aberrant large and giant cells and multinucleated cells) and presence of large acellular spaces were observed in samples of treated mice, in comparison to non treated mice, for which those characteristics were not observed. In Figure 6, examples of histopathological preparations with Ki67 immunostaining, corresponding to green pixels and red pixels areas in tumors, are shown for representative cases of each class (C1110, C979, C971 and C1108). Ki67 immunostainings and nosological images of remaining cases evaluated by histopathology, C1111, C1022, C1026 and C1100, are shown in Figure S5.

**Figure 7:**
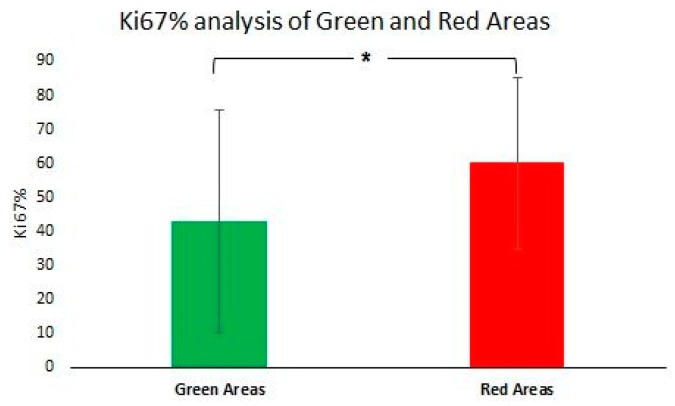
Graph bar of mean ± SD of Ki67% values found in green and red areas from nosological images of all studied cases. Significant differences (*p* < 0.05 with Mann–Whitney’s U test) were found between them, with higher values in red (59.9 ± 25.1%) areas in comparison with green areas (42.9 ± 32.6%).

**Figure 8:**
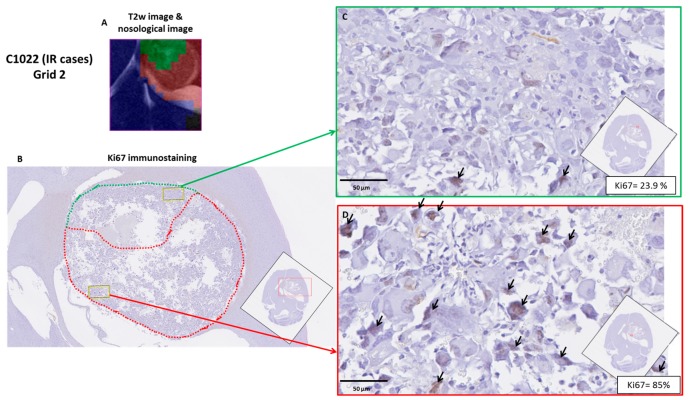
Example of the histopathological validation of a nosological image of Grid 2 of the case C1022 (IR case). (**A**) Nosological image obtained from Grid 2 of the case C1022 superimposed to the T_2w_-MRI. Both green and red zones could be distinguished within the tumor, showing a heterogeneous pattern of response. (**B**) Ki67 immunostaining colocalized with the Grid 2, in which the red and green areas from the nososological image have been manually drawn over the tumor (shown in red and green dashed lines). One representative field has been selected in each area (yellow rectangles have the same area). (**C**) Magnification (40×) of the yellow field from the green area, with an average Ki67% value of 23.9%, and black arrows pointing to Ki67 positive cells. (**D**) Magnification (40×) of the yellow field from the red area, with a Ki67% value of 85%, and black arrows pointing to Ki67 positive cells.

Ki67 analysis: *Comparison of responding and non-responding zones*: green and red areas of the nosological images, disregarding the case categorization, showed a trend, with green zones having lower Ki67 values (42.9 ± 32.6%, *n* = 50 fields) than red zones (52.9 ± 25.1%, *n* = 121 fields) (see Figure 7). Significant differences were found with Mann–Whitney’s U test (*p* < 0.05), although there is considerable dispersion within the data. The Ki67% value found for red zones of treated cases was not significantly different from values obtained in red zones of control untreated cases. Analyzing green and red zones in a case-by-case study (i.e., comparing Ki67 for red and green zones within the same case), the obtained results varied depending on the case: IR cases presented either statistical significance (*p* < 0.05, C1022) (Figure 8) or a trend towards significance (0.15 > *p* > 0.05, 1026, C971), whereas HR cases did not present statistical significance.

Taking into account these results, we wondered whether green and red zones could have significantly different Ki67 values depending on the classification of the case according to *TRI* level (e.g., all green zones of IR cases vs. all green zones of HR cases). For IR cases, we found Ki67 values of 52.0% for red zones and 21.1% for green zones, whereas cases classified as HR presented values of 84.1% for red zones and 77.7% for green zones. This would suggest that although there is indeed a correlation between a responding/non-responding pattern and its Ki67 immunostaining, the absolute values could be quite different between intermediate response and high response cases, which could be at least partially explained due to different cellularity between these cases.

*The global Ki67% for each case* was also analyzed taking into account the Ki67 values found for all slices of a given case, which would reflect an average proliferative state of the whole tumor. Global Ki67 data are shown in Table 2. Still, higher global values were observed in control cases than in treated cases as a whole (Figure S6A, 68.6%, *n* = 40 fields vs. 52.6%, *n* = 139 fields respectively). Significant differences were found with Mann–Whitney’s U test (*p* < 0.05). Regarding treated cases, higher global Ki67 values were observed in LR cases (64.8 ± 7.3%) than in IR cases (41.9 ± 32.2%), as expected (Figure S6B). However, surprisingly, HR cases presented a value higher than expected and a possible explanation, which will be further elaborated in the Discussion Section, can be related to the lower cellularity observed in these cases and the different cell volume observed in treated cases with intermediate or high response, as mentioned before.

**Figure 9:**
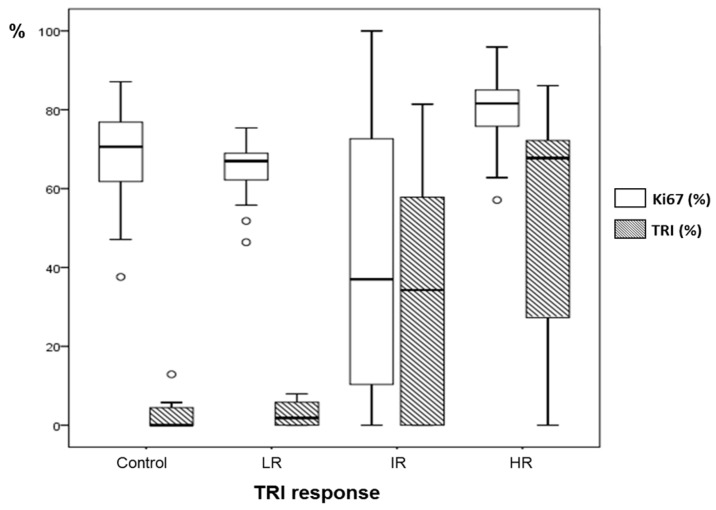
Boxplot of global percentage of Ki67 (white boxes) and percentage of *TRI* values (striped boxes) of each MRSI grid of different groups of cases classified by *TRI* response and analyzed by histopathology. Control and LR cases with low values of *TRI* (2.7 ± 1.1% for control cases and 3.3 ± 0% for LR cases) showed higher Ki67 values (71.6 ± 10.9% for control cases and 64.8 ± 0% for LR cases). IR cases showed similar mean values of *TRI* 42.1 ± 5.6% and Ki67: 43.5 ± 22.0%, presenting also a very high dispersion as well. HR cases showed higher values of *TRI* (as expected, 68.3 ± 2.8%) but unexpectedly high Ki67 values (77.8 ± 6.3%). Boxplot: The limits of the box represent quartiles 1 (Q1) and 3 (Q3) of the distribution, the central line corresponds to the median (quartile 2). The whiskers symbolize the maximum and minimum values in each distribution. Outliers (values higher than 1.5 × IQR, interquartile range, obtained by difference of Q3 and Q1) are represented with round symbols.

Significant differences were observed for Ki67 (*p* < 0.05) between groups of cases classified by their *TRI* response level (LR cases, IR cases, and IR cases) (Figure S6B). The Ki67% of the different groups classified by *TRI* and their *TRI* values are also summarized in Figure 9.

## 3. Discussion

### 3.1. Multi-Slice MRSI and TRI for Therapy Response Level Evaluation

MRSI acquisitions allowed us to obtain differential metabolomic patterns from treated and control tumors. Although it is true that other authors have also applied MRSI techniques in order to assess the effect of therapy in brain tumors [7,8,33,34], they were mainly focused in some particular signals or ratios (such as quantitation of NAA, Cho, Cr or ratios between them). These ratios have proved useful in our model [18] only when using single voxel MRS data, but did not succeed to produce robust discrimination when individual spectra from MRSI grids classified as responding and non-responding were used for the calculation (data not shown). This probably suggests that there is not a single change or a set of few changes/ratios which could fully distinguish a responding from a non-responding zone. In this sense, the source-based approach technique applied to MRSI data in this study takes into account the whole set of possible metabolic changes in the spectral pattern and can handle the information in a way that may not be straightforward to perform after standard quantification, for example with the LCModel [35]. Table 1 lists the main metabolites, lipids and macromolecules thought to contribute to the spectral patterns recorded. The detection of signals at 2.8 ppm, compatible with presence of PUFA is in agreement with authors in [30], who reported similar signals in their preclinical model, as early as two days after therapy with ganciclovir, being indicative of apoptosis.

After acquiring MRSI data from our mice, nosological images were then generated to follow-up the evolution of tumor response to treatment. Previous studies from our group using MRSI-based source analysis from a single slice allowed us to distinguish between responding and non-responding cases [13], with metabolomics pattern derived changes preceding the anatomical derived information provided by MRI in several instances. However, the use of a single slice, ignoring the response level taking place in other tumor areas, could prevent us to gather relevant information, as GBM is widely accepted to be highly heterogeneous [36]. The multi-slice MRSI technique used in this study has allowed us to obtain nosological images from different areas of the investigated tumors, unraveling heterogeneous response levels that we were unable to see with the single slice examination. In work presented in [13], several treated cases presented a response level (when recalculated for the presently proposed *TRI* index) close to 100% (mean *TRI* = 92.9 ± 8.2%, *n* = 8) whereas in the present work with whole tumor examination, the maximum value achieved was of 70.3% and the mean ± SD of all treated mice was 40.8 ± 20.5%. Still, we observed that not all MRSI slices had the same response level after treating mice with TMZ, with upper more dorsal slices presenting higher levels of *TRI* (53.0 ± 28.4%) than lower more ventral ones (8.6 ± 20.0%) (*p* < 0.05). Once the *TRI* parameter was calculated, it allowed us to quantify the evolution of response to treatment and also to classify cases in different arbitrary categories. It is worth noting that the generation of nosological images presents some limitations, namely: highly heterogeneous or hemorrhagic zones could result in poor quality MRSI spectra, preventing the system to classify them correctly. In addition, the smallest tumor correctly segmented had a volume of 18.4 mm^3^, which should be taken into account when interpreting data of apparently non-responding or normal parenchyma regions.

### 3.2. Classification of TMZ-Treated Mice after TRI Calculation and Evolution of TRI Values

Treated mice were classified into different groups, as explained in results Section 2.1 and Section 2.2 and Table 2. The first part of the study only produced LR and IR cases, and a modification of the initial protocol was needed in order to obtain cases fulfilling preset criteria for HR cases. The fact that some cases presented only partial response to therapy was seen in our previous work [13] with one single MRSI slice in cases C418, C527 and C584 (with *TRI* response now calculated for those single-slice acquisitions of 21.3%, 60.6% and 49.0%, respectively). However, the detailed subdivision in “response groups” and heterogeneity observation was only possible after performing a multi-slice MRSI acquisition, confirming the heterogeneous pattern of response usually seen in GBM. This heterogeneous pattern of response has also been reported in other preclinical studies using diffusion-weighted MRI techniques [37] and using PET-MRI techniques in humans [38].

The evolution of *TRI* in the different cases studied with longitudinal measurements indicated that increases in *TRI* corresponded essentially to periods of growth arrest (stable disease stage following RECIST criteria) and following therapy cycles. Regarding IR cases, the *TRI* increase was seen around 6.5 ± 1.0 days after the first therapy cycle (*n* = 4), except in *n* = 2 cases in which the highest increase was seen six days after the second therapy cycle. It is worth mentioning that in IR cases (see Figure 1A,B), the first appearance of the responding pixels preceded the growth arrest observed in MRI acquisitions. On the other hand, LR cases presented a slightly different evolution: these cases did not show tumor growth arrest even when they were treated and *TRI* pixels were first observed during the first therapy cycle (earlier than in IR cases, see Figure 1C,D). Still, some cases, i.e., C971, seemed to show an oscillating pattern for *TRI* (see Figure 1A) in which two peaks of increased *TRI* (31.2% and 44.1%) were separated by eight days.

The protocol designed for this study was focused in allowing the histopathological validation of nosological images recorded at certain time points, meaning that mice fulfilling criteria for inclusion in a response group were euthanized after MRSI acquisition to allow for in vivo/in vitro correlation. Accordingly, we could not observe the evolution of *TRI* along the whole treatment period for the investigated mice. To avoid this restriction, one of those mice (C974) was saved to be followed up until end-point. In this mouse we could clearly observe a cyclical pattern in *TRI* values, as opposed to tumor volume, which remained stable during six days (maximum variation of 11.20% corresponding to stable disease following RECIST criteria). The *TRI* presented different values along tumor evolution (see Figure 2), with the first maximum peak of *TRI* (41.4%) being observed at Day 20 p.i. (five days after finishing the first therapy cycle), whereas the second maximum peak of *TRI* (36.8%) was observed at Day 26 p.i. (six days after finishing the second therapy cycle). This cyclical observation of *TRI* peaks (5–6 days after therapy cycles) was taking place at the same time that tumor volume was indicating “stable disease” according to RECIST criteria. Then, a last peak of *TRI* was observed at Day 37 p.i., when tumor relapsed (at progressive disease stage). This was 12 days after finishing the last therapy cycle and the animal died due to tumor mass effect (140.5 mm^3^). As the objective in this case was not focused in histopathological validation, no samples of C974 were saved for such analysis.

This cyclical pattern of response was not obvious in other IR cases (C975, C1022, C1023 and C1026), in which *TRI* increases were observed during tumor growth arrest, or when tumor growth arrest started to be observed. However, it is possible that the prompt animal euthanization, when these *TRI* increases were observed, could have prevented us to observe further *TRI* oscillations.

The HR cases were only observed when the protocol was adjusted to start therapy with smaller tumor volumes (3.7 ± 0.8 mm^3^). In these, *TRI* increase was observed five days after the second therapy cycle, although in these cases the MRSI acquisition was conditioned to tumor volume decrease and no previous MRSI data was acquired. Finally, LR cases did not respond to therapy and no *TRI* oscillations were observed. The reason why these cases were not responding could be due, among other reasons, to individual tumor resistance to alkylating agents, which is mainly due to overexpression of the MGMT protein, and it is a major and currently unresolved problem with TMZ therapy [39]. Still, different glioma-initiating cells (GIC) clones from the same GBM tumor may have different levels of therapy resistance to different therapies, resulting in a heterogeneous pattern of response to therapy in the same tumor [36]. It was beyond the scope of our present study to establish whether a correlation between *TRI* level and overall survival exists or not. However, from the data gathered, it was clear that the behavior of *TRI*, as opposed to the relatively unchanged tumor volume during growth arrest, presented an oscillating pattern with an approximated frequency around 6.3 ± 1.3 days (*n* = 4). See possible explanations for this in Section 3.4.

Intuitive cyclical changes in *TRI* at stable disease stages could be observed in three longitudinal cases included in [13]. These cases (C817, C819 and C821) were followed up until endpoint by single slice MRSI. The mean frequency of these *TRI* cycles now recalculated (6.3 ± 2.0 days, *n* = 3) was very similar to the one calculated using the multi-slice approach in this work (6.3 ± 1.3 days). Nonetheless, the representation of *TRI* evolution is clearer in cases studied by the multi-slice approach than in single slice MRSI cases.

### 3.3. Histopathology Results

Histopathology analysis was conducted for all acquired grids in chosen animals (see Section 2.4) in order to confirm the results previously described in [13], in which an inverse and significant correlation between the responding pattern and the proliferation marker Ki67 was found, with green, responding zones presenting a significantly lower Ki67% value. This histopathological biomarker was also chosen by other authors in order to estimate therapy response in preclinical brain tumors [40,41] and in human glioblastoma [42,43].

As can be seen in Figure 7, this trend has been also seen in this work with multi-slice MRSI acquisition, although the fold change was slightly different (1.4 fold change in this study, compared to 2.4 fold change obtained in previous studies of our group with one single MRSI slice [13], when comparing zones with red or green pattern). Although the dispersion of values was really large, especially within green zones, we have been able to confirm that lower mean values of Ki67 are found in green zones, whereas higher values are seen in red zones, either from controls or treated tumors.

One of the unexpected findings in this work was that HR cases had the highest calculated Ki67% values, in disagreement with tumor volume behavior, which was showing signs of volume decrease. The lower cellularity in investigated fields from those cases, with large acellular spaces between abnormal giant cells (morphology described by other authors in human glioma treated with radiotherapy [44] and in human treated sarcoma [45]) could be partially responsible for that. We wondered if analyzing the number of Ki67 positive cells by mm^2^ instead of percent tumor cells would be helpful for clarification, and the results may be seen in Figure S7, in which the number of Ki67 positive cells/mm^2^ is illustrated. A descending trend is clearly seen between control (2912.4 ± 692.7 cells/mm^2^, *n* = 2), LR (1799.2 ± 369.7 cells/mm^2^, *n* = 1) and IR (372.7 ± 264.7 cells/mm^2^, *n* = 3) cases. Regarding HR cases, values found (713.6 ± 211.5 cells/mm^2^, *n* = 2) were slightly higher than IR cases, but lower than LR and control cases. In addition, significant differences were found with Mann–Whitney’s U test (*p* < 0.05) between the groups. This would probably suggest that the absolute number of Ki67 positive cells for mm^2^ would not be quite different in IR and HR cases, but the overall abnormal morphology of the cells as well as tumor behavior and the metabolomics pattern from MRSI acquisitions are indicating that the response level is higher in HR cases.

Having in mind the different morphology observed in cases under treatment and the effect that it seemed to produce in Ki67% calculation, we tried to assess the mean cell volume in each group of cases, as well as the amount of acellular space observed in histopathological slides in order to better understand which kind of elements would be essentially contributing to the observed MRSI pattern. For this, we calculated the average volume of Ki67 positive cells present in a representative field of different cases (*n* = 8 cases, 2 HR, 1 LR, 3 IR and 2 control) (Figure S8A) as well as the percentage of acellular space observed (in the same group of cases) (Figure S8B). In Figure S8A, an ascending trend of increasing cell volume is observed: control cases (372.1 ± 178.8 µm^3^, *n* = 50 cells counted), in LR cases (1867.3 ± 1698.9 µm^3^, *n* = 25 cells), in IR cases (6179.9 ± 9855.5 µm^3^, *n* = 54 cells) and in HR cases (8180.6 ± 7608.3 µm^3^, *n* = 45 cells). Significant differences were found with Mann–Whitney’s U test (*p* < 0.05) between the groups. This could probably distort the Ki67% estimation value if the calculations are done in the same way for control and responding tumors, because the average cell volume could be 22 fold higher in an HR case than in a control case, and a given field, accordingly, will be represented by a lower number of cells. Furthermore, a high number of histological fields with plenty of acellular space was observed in treated cases, which cannot be neglected either. Regarding them, in figure S8B an increasing trend in the percentage of acellular space is observed: control cases presented only 19.1 ± 6.9%, whereas ascending values were observed in treated cases: LR cases with 27.5 ± 8.2%, IR cases with 37.4 ± 11.0% and HR cases 44 ± 15.3% which is almost half of the field. Significant differences were found with Mann–Whitney’s U test (*p* < 0.05) between groups.

These acellular spaces are large, irregular and they are usually observed in the central part of treated tumors, which indicates that they were caused by therapy with temozolomide and not by tissue fixation and processing. In the case of acellular spaces due to tissue fixation, those spaces are usually observed in the tumor periphery at the border zone between tumor and normal brain parenchyma.

Therefore, control (untreated) cases showed smaller cell volumes of Ki67 positive stained cells as well as much less acellular space than treated cases, resulting in fields with high average cellularity. On the other hand, in treated cases, the increase in response level (*TRI* values) was accompanied by an increase in cell volume (with presence of giant cells) and a higher presence of acellular space in histological fields. Accordingly, the difference in cell volume and percentage of acellular space in the evaluated histopathological fields could partially explain why an apparently high Ki67 value is found in HR cases if a standard percent counting is performed. It may be more adequate to analyze results the way shown in Figure S7, for which the number of Ki67 stained cells is seen to significantly decrease between control, LR and IR + HR cases.

**Figure 10:**
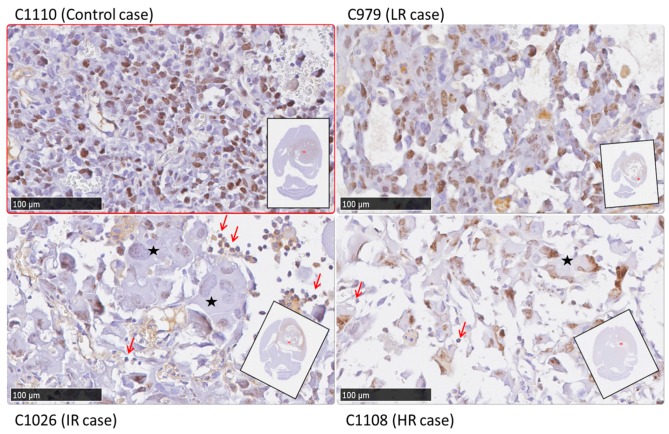
Histopathological regions (20× magnification) from representative fields of control, LR, IR and HR cases. A higher number of uniform and small tumoral cells were observed in the control case in comparison to LR, IR and HR cases. A low number of large tumoral cells were observed in treated cases (with many of them possibly converting to giant cells in IR and HR cases). Regarding acellular spaces, in control cases, cells were more densely organized and few such spaces were observed. On the other hand, in treated cases, especially HR cases, the percentage of acellular space was significantly higher (see also Figure S8). In IR and HR cases, numerous lymphocyte-like cells (red arrows) and giant cells (black stars) were also observed.

To illustrate data used to calculate Figure S8, representative fields of control, LR, IR and HR cases are shown in Figure 10. In control cases, uniform smaller cells and fewer acellular spaces were observed than in treated cases. In those treated cases, with higher *TRI* response levels, cell morphology changed: giant cells were observed as well as an increase in acellular space (white spaces shown in Figure 10) was detected.

An interesting additional finding was that in IR and HR cases, small lymphocyte-like cells were observed (red arrows in Figure 10), which would be compatible with tumor infiltration by lymphocytes, although further confirmatory work will be needed for a full validation of the extent of this observation. These lymphocyte-like cells were not observed in LR and control cases.

It is worth stressing that obtaining HR cases was not straightforward and required modifications of the initially designed protocol. Even when modifications were introduced, and this is one of the important findings in this study, the metabolomics based analysis of multi-slice MRSI showed that there were in our hands no 100% responding tumors, reinforcing the idea that GBMs present high heterogeneity, probably with clonal selection of resistant cells after therapy administration. The use of combined therapeutic agents, as well as modified administration schedules such as metronomic administration, could be of help, as promising results have been recently obtained in our group with the same preclinical GBM model [46].

In this multi-slice study, in which a tridimensional-like reconstruction approach was attempted, it was possible to establish a relationship between groups of cases with different *TRI* levels and Ki67 values. The global analysis case by case of multi-slice acquisitions can provide a good estimation of the response level of the whole tumor probably better than using the single slice approach, which could provide an overoptimistic outcome. The reason for this being that the slice used for single slice protocol usually corresponded to the Grid 2 in the multi-slice protocol, and as previously stated, most of the green pixels, corresponding to the responding pattern were located in Grids 1 and 2. In this sense, the multi-slice approach provided a more realistic analysis while the inverse correlation found between *TRI* and the Ki67 “global” value (see for example Figures S6 and S7) confirmed the findings from previous work of our group [13].

### 3.4. A Possible Explanation for the Cyclical TRI Behavior: TMZ Therapy Triggering Immune Response in Host

**Figure 11:**
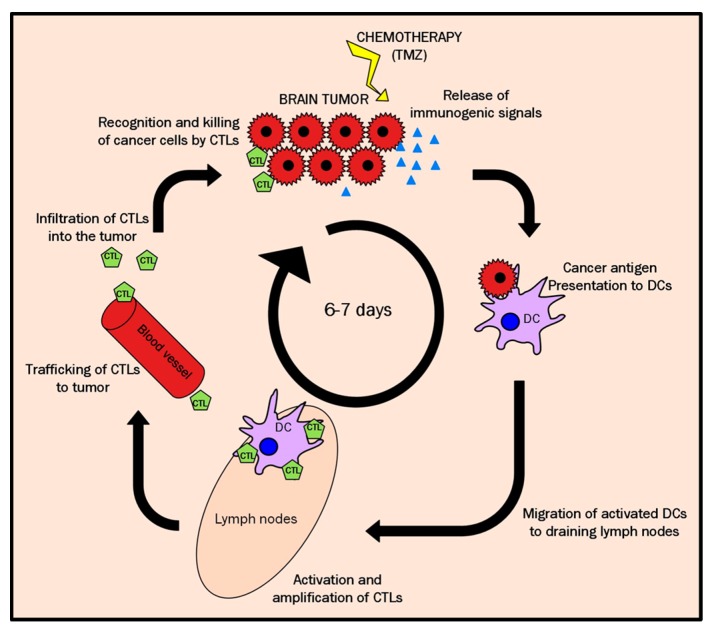
Scheme of the cycle for immune response against a brain tumor. The whole cycle in mouse brain is assumed to take around 6–7 days [49]. The malignant cells may release immunogenic signals, after the chemotherapeutic agent (TMZ) effect on them. These signals attract dendritic cells (DCs) precursors to the tumor, were they are activated by compromised tumor cells. Then, activated DCs migrate to local lymph nodes (LN), like dorsal cervical lymph node for brain, and present processed tumor-derived peptides to naïve T and B-lymphocytes. In case of antigen match, they are then activated to become plasma cells producing antibodies, CD4^+^ helper T lymphocytes or CD8^+^ cytotoxic T lymphocytes (CTLs). Activated CTLs leave LN to infiltrate brain tumor tissues and exert effector functions [50,51].

From previous and present data from our group, *TRI* has been proven to follow a non-linear behavior, with an oscillating pattern, which was intuitive in single-slice cases from [13] but became clear in the multi-slice recordings of mice in this work. This is suggesting that the metabolomic pattern changes are dynamic and vary along time. They not only vary among different cases, but they vary inside the same tumor as well, indicating a differential behavior which seems to display an apparent frequency of ca. 5–7 days. One of the possible explanations for that lies in the mechanism of cell damage triggered by TMZ. Some authors have described that after alkylating agents (e.g., TMZ) treatment a recruitment of the host immune system takes place [47,48] which is relevant in the response to therapy, eventually triggering tumor cell death. Still, it is described in [49] that the whole immune cycle (see Figure 11) in mice brain usually requires around six days, which agrees with the oscillation period recorded in this work (see Figure 2). The process includes recognition of local antigens by dendritic cells (DC), migration to regional lymph nodes, where they present processed tumor-derived peptides to naïve CD8^+^ and CD4^+^ T cells, which leave afterwards lymphoid organs to infiltrate tumor tissues and exert effector functions, killing tumor cells.

All this taken into account suggests that the metabolomics responding pattern could act as a surrogate biomarker of these immune cell “waves” killing sensitive tumor cells. Preliminary findings of infiltrative cells compatible with lymphocytes (Figure 10), seen in IR and HR cases, would agree with this interpretation. Nevertheless, further histopathological validation will be needed to fully confirm the identity of these cells and their possible contribution to the recorded metabolic pattern. The rich information contained in these oscillatory metabolomics pattern changes is discrepant with the lack of tumor volume changes during the growth arrest phase. New “repopulation” of the tumor mass with actively proliferating GL261 cells with new clonal characteristics may cause the oscillating peaks of non-responding tumor (red pixels). The initially activated CD8^+^ T cells would not be effective against those new sub-clones of GL261 cells and another cycle of DC priming and new CTL clones activation would be required.

The participation of the immune system in the response to therapy has been widely described by different authors [52,53] and GBMs are known to exhibit varying degrees of infiltration with mononuclear cells, consisting primarily of T lymphocytes, reinforcing the idea of a cell-mediated immune response [54] although these tumors are also known to secrete a number of immunosuppressive factors. This intratumoral infiltration with CD4^+^ and CD8^+^ T cells was also seen in preclinical glioblastoma (GL26 glioma-bearing C57BL/6 mice) after immunomodulatory treatment [55]. The described GBM infiltration with T lymphocytes, on the other hand, could be a likely explanation for the appearance of green, responding pixels even in nosological images of mice which did not receive treatment.

Other evidences also support the hypothesis of the immune system participation in response to therapy. For example, the use of a metronomic scheme of therapy administration, optimized in order to activate host immune responses, has been used with excellent results in ectopic GL261 preclinical models [48] even producing the cure of animals, as well as inducing long-term immune memory against the tumor. Authors in [48] used a six-day interleave period for therapy administration (cyclophosphamide), demostrating that this interval was optimal in their case for improving the immune system participation. Metronomic administration, usually referring to administrations of low and equally spaced doses of chemotherapeutics without long rest periods in between [47,48] has been used to improve immune responses to potentiate tumor regression and avoid regrowth [48]. Our group has also used this scheme in work reported in [46], obtaining a survival time slightly higher (38.7 ± 2.7 days, *n* = 6) than the traditional scheme with three cycles [18] used in the present work (5-2-2 days with a three-day interleave between them (33.9 ± 11.7 days, *n* = 38)). Still, work reported in [46] highlighted the relevance of the metronomic strategy in comparison with traditional administration schemes in which results suggested an impairment of the immune system related response due to continuous therapy administration, probably interfering with T cell amplification.

However, it is important to remark that the 5-2-2 cycle therapy does not follow a metronomic schedule, although data in Figure 2 clearly show a close to six-day oscillation of “responding pixels” percentage. However, it is true that the 5-2-2 protocol does not follow a metronomic scheme, data gathered in [18] showed that a single cycle of TMZ during five days did not produce any improvement in animal survival (20.6 ± 6.8 days for 1 TMZ cycle vs. 20.5 ± 4.1 days for control animals). It was only when the two additional cycles were introduced with a three-day interleave between them that the survival rate increased significantly to 33.8 ± 8.7 days. The interleave between the middle time point of each cycle is, in the 5-2-2 protocol, six days between the first and second cycle, and five days between the second and third cycle, which could have contributed to configure a “metronomic-like” therapeutic scheme, favoring the host immune system participation in response to therapy also in our present study. Nevertheless, it is also possible that the duration of the first TMZ cycle (five days) partially prevented the full immune cycle recruiting, with a balance between favorable and unfavorable conditions. The sub-optimal length of TMZ administration could trigger cell damage, both in tumor cells but also in proliferating primed CD8^+^ lymphocytes in the lymph nodes, which should later infiltrate the tumor. Accordingly, the second “response” peak (seen after the second therapy cycle in Figure 2) would be a mixture of new immune system attraction through immunogenic signaling caused by the previous TMZ treatment, and a wave of the remaining CD8^+^ lymphocytes not compromised by TMZ administration. Further work will be needed in this sense: if the 6–7-day cycle for immune system activation is proven correct in our GBM preclinical system, an adjustment of cycles should be done and the interleave between the first and second cycles should be 6–7 days for future work, as well as a shortening of the first cycle to a single administration to avoid interfering with T-cell amplification.

In addition, in order to fully characterize the immune system cells differentially present in responding and non-responding zones (red and green patterns in nosological imaging), additional immunostaining methods may be applied, such as CD8a and foxP3 as described in [56]. This would be of help to better understand potential contributors to the metabolomics pattern of responding and non-responding tumor zones. Last, but not least, the nosological images here described could be of relevance to design personalized therapeutic schemes, being able to detect when a tumor starts failing to respond to a first line therapy, providing a time frame for considering a second line or a combination therapy approach.

## 4. Materials and Methods

### 4.1. GL261 Cells

GL261 mouse glioma cells were obtained from the Tumour Bank Repository at the National Cancer Institute (Frederick, MD, USA) and were grown as previously described [27].

### 4.2. Preclinical Glioblastoma Model for in vivo Studies

The use of animals in this study has been reported according to the ARRIVE guidelines [57].

Animals were obtained from Charles River Laboratories (L’Abresle, France) and housed in the animal facility of the *Universitat Autònoma de Barcelona*. All mice described in this work are identified with a unique alphanumeric identifier of the type CXXXX. Tumors were induced in a total of 39 C57BL/6 female wild-type (wt) mice (weighing 21.1 ± 1.3 g) by intracranial stereotactic injection of 10^5^ GL261 cells as previously described by us [26], although not all of them were selected for therapy administration (final number of included animals *n* = 26). In addition, not all animals receiving therapy met criteria for inclusion in classes described in Section 4.5, resulting in a final number of studied animals being lower than the initial number of generated animals.

Still, two different groups of animals were produced:

Group A: Tumors were generated in different series of animals separately in time for proper schedule allocation in the NMR facility. In the first part of the study, 5–7 animals were injected in each cell-injection series. Mice were weighed every day and tumor volumes were followed twice or three times a week using T_2_-weighted MRI acquisition (welfare parameters were followed up as explained in Table S2). From the animals injected in each series, the three mice with the most homogeneous weights and tumor sizes were chosen for the multi-slice MRSI experiment before starting therapy.

Group B: In the second part of the study, tumors were induced in 19 mice. Four of them were discarded because no tumor was detected after 10 days p.i. Regarding the remaining 15 mice, they were weighed every day and tumor volumes were followed twice or three times a week using T_2_-weighted MRI acquisition in all generated tumor-bearing animals. However, only mice showing tumor volume decrease after therapy (see next section for therapy details), in comparison with previous measurement, were selected for multi-slice MRSI experiments.

In addition, four control (non treated) tumor-bearing mice were also explored by multi-slice MRSI.

### 4.3. Animal Treatment with Temozolomide

TMZ (*Sigma-Aldrich, Madrid, Spain*) was dissolved in 10% DMSO in saline solution (0.9% NaCl) for in vivo experiments and it was administered using an oral gavage at a dose of 60 mg/kg as in [18].

In the first part of the study (group A), therapy administration started at Day 11 p.i. (tumor volume average 7.5 ± 5.0 mm^3^) with 3 cycles of therapy administration as previously described by our group [18]. Schema of TMZ administration is shown in Figure S9. In the second part of the study (group B), the therapy starting day was not always the same, being adapted to start when the tumor volume was between 3 and 5 mm^3^ ± 10%.

All studies were approved by the local ethics committee (*Comissió d’Ètica en Experimentació Animal i Humana* (CEEAH), http://www.recerca.uab.es/ceeah accessed on 16 November 2016), according to the regional and state legislation (protocol DMAH-8236/CEEAH-2785).

### 4.4. In vivo MRI and MRSI Studies

#### 4.4.1. Data Acquisition

In vivo MRI/MRSI studies were performed at the joint nuclear magnetic resonance facility of the *Universitat Autònoma de Barcelona* and *Centro de Investigación Biomédica en Red—Bioingeniería, Biomateriales y Nanomedicina* (CIBER-BBN) (*Cerdanyola del Vallès*, Spain), Unit 25 of NANBIOSIS. The 7T Bruker BioSpec 70/30 USR spectrometer (Bruker BioSpin GmbH, Ettlingen, Germany) equipped with a mini-imaging gradient set (400 mT/m) was used. A 72 mm inner diameter linear volume coil was used as transmitter, and a mouse brain surface coil as a receiver for brain MRI studies.

Mice were positioned in a bed, which allowed delivery of anaesthesia (isoflurane, 1.5–2.0% in O_2_ at 1 L/min), with an integrated heating water circuit for body temperature regulation. Respiratory frequency was monitored with a pressure probe and kept between 60–80 breaths/min.

●  MRI studies

GL261 tumor-bearing mice were screened by acquiring high resolution coronal T_2w_ images using a Rapid Acquisition with Relaxation Enhancement (RARE) sequence to detect brain tumor presence and to monitor its evolution stage. The acquisition parameters were as follows: repetition time (TR)/effective echo time (TE_eff_ ) = 4200/36 ms; echo train length (ETL) = 8; field of view (FOV) = 19.2 × 19.2 mm; matrix size (MTX) = 256 × 256 (75 × 75 μm/pixel); number of slices (NS) = 10; slice thickness (ST) = 0.5 mm; inter-ST = 0.1 mm; number of averages (NA) = 4; total acquisition time (TAT) = 6 min and 43 s.

MRI data were acquired and processed on a Linux computer using ParaVision 5.1 software (Bruker BioSpin GmbH, Ettlingen, Germany).

●  MRSI studies

**Figure 12:**
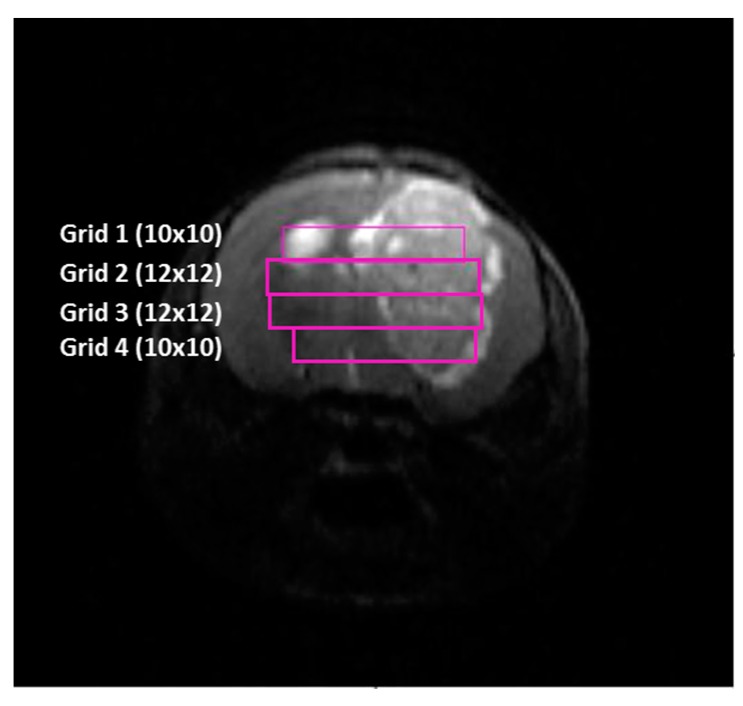
Coronal T_2w_ MRI of mouse C971 brain, with the position of the four MRSI slices.

Consecutive 14 ms TE MRSI with PRESS localization grids were acquired individually across the tumor, using as a reference T_2w_ high resolution images, as shown in Figure 12. First upper (dorsal) grid (Grid 1) had a matrix size of 10 × 10. Then, Grid 2 was acquired 1 mm below Grid 1 with a matrix size of 12 × 12. Grid 3 was acquired 1 mm below Grid 2, with a matrix size of 12 × 12. Finally, if tumor volume was not completely covered with 3 grids, a last Grid 4 was acquired 1mm below Grid 3 with a matrix size of 10 × 10. In order to ensure quality of the acquired data, shimming was performed individually for each MRSI grid. MRSI grids were spatially located such that the volume of interest (VOI) included most of the tumoral mass as well as normal/peritumoral brain parenchyma.

Acquisition parameters for all grids were: FOV, 17.6 mm × 17.6 mm; VOI in Grids 1 and 4 was 5.5 mm × 5.5 mm × 1.0 mm. VOI in Grids 2 and 3 was 6.6 mm × 6.6 mm × 1.0 mm. ST, 1 mm; TR, 2500 ms; Sweep Width (SW), 4006.41 Hz; NA, 512; TAT, 21 min 30 s. Water suppression was performed with Variable Power and Optimized Relaxation Delay (VAPOR), using a 300 Hz bandwidth. Linear and second order shims were automatically adjusted with Fast Automatic Shimming Technique by Mapping Along Projections (FASTMAP) in a 5.8 mm × 5.8 mm × 5.8 mm volume which contained the VOI region. Six saturation slices (ST, 10 mm; sech-shaped pulses: 1.0 ms/20250 Hz) were positioned around the VOI to minimize outer volume contamination in the signals obtained. Total acquisition time for a typical MRI/MRSI 4 grids full protocol was about 3.5 h.

In the first part of the study, MRSI experiments were acquired every 2–3 days, from the day before starting therapy until animal euthanization. However, in the second part of the study, MRSI experiments were not acquired from the beginning of therapy administration. Tumor volume was followed up by MRI and when tumor size was reduced, compared to the volume observed in the previous MRI measurement, MRSI studies started to be carried out every 2–3 days until animal euthanization.

#### 4.4.2. MRI and MRSI Processing and Post Processing

●  Volume calculation

Manual segmentation of abnormal brain mass in T_2w_ images was performed by LF, and tumor volumes were calculated from T_2w_ high resolution horizontal images using the equation:

(1) $$TV\;\left(mm3\right)=\left[\left(AS_{1}\times ST\right)+\;\left[\left(AS_{2}+\;\left(\ldots \right)+\;AS_{10}\right)\;\times \;\left(ST+IT\right)\right]\right]\;\times 0.075^{2}$$

where *TV* is the tumor volume, *AS* is the number of pixels contained in the region of interest delimited by the tumor boundaries in each slice of the MRI sequence, *ST* is the slice thickness (0.5 mm), *IT* the inter-slice thickness (0.1 mm) and 0.075^2^ mm^2^ the individual pixel surface area. The tumor area was calculated from pixels in each slice, using an automated system for generating regions of interest (ROIs) available in the ParaVision 5.1 software (Bruker BioSpin, Ettlingen, Germany). The inter-slice volume was not registered and it was estimated adding the inter-slice thickness to the corresponding slice thickness in Equation (1).

●  Brain MRSI post-processing and pattern recognition (PR) strategies

MRSI data were post-processed essentially as described in [27]. Briefly, data were initially pre-processed at the MR workstation with ParaVision 5.1 (Bruker BioSpin), and then post-processed with 3D Interactive Chemical Shift Imaging (3DiCSI) software package version 1.9.17 (Courtesy of Truman Brown, Ph.D., Columbia University, New York, NY, USA) for line broadening adjustment (Lorentzian filter, 4 Hz), zero-order phase correction and exporting the data in ASCII format. Dynamic MRSI processing Module (DMPM), running over MatLab 2013a (The MathWorks Inc., Natick, MA, USA) was used to align all spectra within each MRSI matrix (using the choline containing compounds peak as reference, 3.21 ppm). The 0–4.5 ppm region of each spectrum in the MRSI matrix was individually normalized to UL2 and the normalized matrix was exported in ASCII format for performing the PR analysis. No baseline correction was performed in these spectra.

After that, the NMF semi-supervised methodology [58,59] was applied for the extraction of meaningful source signals from the MRSI investigated tumors. In general, non-negative matrix factorization (NMF) methods belong to a group of multivariate data analysis techniques designed to estimate meaningful latent components, also known as sources, from non-negative data. Standard NMF methods decompose a given data matrix “**X**” into two non-negative matrices: the sources (“**S**”) and the mixing matrix (“**A**”). The differences between these NMF methods are given by the different cost functions used for measuring the divergence between **X** and S*A. In addition, convex NMF, used in this work, is also able to handle negative data [59,60].

From the biochemical viewpoint, the source extraction technique to classify MRS data assumes that in each voxel there is a mixture of heterogeneous tissues and its metabolites from which the contribution of each source can be obtained. A previously described semi-supervised approach [13], based on cNMF for initial source extraction, was used for classifying pixels into normal brain parenchyma, actively proliferating tumor and tumor responding to treatment; and for calculating nosologic maps representing the spatial response to treatment, as in [13]. Green color is used when the GBM responding to treatment source contributes the most, blue for normal brain parenchyma, red for actively proliferating GBM and black for undetermined tissue.

### 4.5. Tumor Responding Index (TRI) Calculations

In order to measure the level of response to treatment using the obtained nosological images, an arbitrary parameter named *TRI* was estimated (Equation (2)).

(2) $$TRI=\frac{Tumour\;responding\;pixels}{Total\;tumour\;pixels}\;\times 100$$

*TRI* is stated as the percentage of green responding tumor pixels of all grids over the total tumor pixels of all recorded grids. Then, tentative ranges of *TRI* categories were established to classify the different response to treatment levels observed in the studied animals, taking into account both *TRI* percentage and volumetric data from MRI measurements meeting “stable disease” criteria according to RECIST [5].

High response cases (HR): *TRI* > 65%, and also tumor size should be reduced with respect to tumor volume observed in the previous MRI measurement. Green pixels must be observed in at least two consecutive MRSI grids

Intermediate response cases (IR): *TRI* range 35–65%, also tumor size should be unchanged, reduced or not increase more than 20% with respect to tumor volume observed in the previous measurement (response or stable disease stage by RECIST criteria) and green pixels must be observed in at least two consecutive MRSI grids

Low response cases (LR): *TRI* < 35% and also tumor size must be more than 20% larger in comparison with the previous measurement.

#### Animal Euthanasia and Sample Storage

Animals were followed up by MRI-MRSI and when one animal met the requirements to be included in one of the *TRI* categories, this animal was euthanized by cervical dislocation and its brain was resected and saved in paraformaldehyde 4% for histopathological analysis.

### 4.6. Histopathology Studies

Fixed brain was embedded in paraffin and serial horizontal sections were performed to correlate the histological preparations with the reference nosological images from the MRSI acquisition. The upper half of the brain (similar to the positions of MRSI Grid 1 and MRSI Grid 2) was cut in 20 sections of 5 µm, and the lower half of the brain (similar to the positions of MRSI Grids 3 and 4) was also cut in 20 sections of 5 µm.

These sections were analyzed by Hematoxylin-Eosin (HE) staining in order to identify the different cells from normal brain and tumor tissue. Then, one section corresponding to each MRSI grid position was selected for Ki67 (BD Biosciences, Madrid, Spain) immunohistochemical staining for detecting cell proliferation. After immunostaining, the preparations were digitized for quantification using a Nanozoomer 2.0HT (Hamamatsu Photonics France, Massy, France). Then, Ki67 positive cells were counted in each section in areas that corresponded to green or red tumor areas observed in the nosological images. From each area (either green or red), 5 fields of 0.0635 mm^2^ were selected to count Ki67 positive cells at 40× magnification, using NDPview 1.2.53 software (Hamamatsu Photonics France SARL, Massy, France). In all tumors, we have placed the five fields in more highly cellular areas (either green or red), avoiding poor or acellular areas. Exceptionally, in some samples, fewer fields were counted if the corresponding green or red area of the nosological image was not large enough to count 5 fields (mean numbers of fields counted considering all samples: 6.3 ± 2.1 of fields counted per grid)

This counting was carried out by 2 different people: one who established the areas (green or red) to be counted, placing fields in areas where cells were observed, and a second observer, who did not know if the animal was treated one or not, or if the field to be analyzed corresponded to a green or red area. The 2 different observers evaluated in every picture, at the same magnification, the area occupied by cells and acellular spaces, giving a percentage for each parameter.

For Ki67, different approaches of counting were attempted, namely:

Ki67% for each field was calculated as described in Equation (3):

(3) $$\frac{Number\;of\;tumoral\;Ki67\;positive\;cells}{Number\;of\;total\;tumoral\;cells}\;\times \;100$$

Global Ki67% for each case was calculated as the mean Ki67% of all analyzed fields.

Ki67/mm^2^, was calculated for each field as described in Equation (4):

(4) $$\frac{Number\;of\;tumoral\;Ki67\;positive\;cells}{Field\;area\;\left(mm^{2}\right)}$$

where field area was 0.0635 mm^2^ in the studied fields.

Finally, the volume of Ki67 positive cells was calculated through measures of the mean diameter of cells in chosen fields, which was used with the sphere volume formula, assuming cells with a spherical form.

### 4.7. Statistical Analysis

Data was evaluated for compliance with the normal distribution using the Shapiro–Wilk and Kolmogorov–Smirnov tests. The presence of outliers in samples was tested with Grubb’s and Dixon’s tests. Variance homogeneity was evaluated with the Levene’s test. A two-tailed Student’s *t*-test for independent measurements was used for comparisons, for samples of equal or different variances (depending on the Levene’s test result) in case of samples following a normal distribution; and Mann–Whitney’s U test was applied to samples following non-normal distributions. The global evolution of body weight measurements was evaluated with the UNIANOVA test. The significance level for all tests was *p* < 0.05.

## 5. Conclusions

A multi-slice MRSI, non-invasive recording approach is feasible in our preclinical model and may be beneficial to properly evaluate the overall therapy response in preclinical GBM. This approach may be used to generate volumetric nosological maps to follow-up treatment and plan on further therapy. Histopathological studies with global Ki67 confirmed results from previous work from our group in which an inverse correlation is seen between the responding pattern level and Ki67 proliferation rate. Still, we could break down responding cases in different response levels calculated from nosological imaging, which, accordingly, were related to global Ki67 for each of those cases. However, a word of caution should be raised when interpreting Ki67 data from cases with widely different cell morphology and volumes, as this could cause an apparent mismatch between Ki67 and metabolomics data producing nosological images.

The tumor responding index calculated from nosological images showed an oscillating pattern with peak maxima every 6–7 days, which would be in agreement with host immune system recruitment for therapy response in our experimental GBM model. This is in agreement with histopathological findings of lymphocyte-like cells infiltrating IR and HR cases but not observed in LR and control cases. Further work on infiltrating immune cells characterization will be needed to confirm or discard this hypothesis.

## Figures and Tables

**Table 1:** List of the main metabolites contributing to the spectral pattern recorded in tumor/peritumoral MRSI of responding and non-responding mice, with the corresponding approximate ppm position. Major contributions are shown in italic, although other signals can also be identified and are also contributing to the recorded pattern. Metabolite assignments according to [18,22,23,24,25,26,27,28,29,30,31,32].

Metabolite	ppm
*Mobile Lipids + Macromolecules*	*0.90*
*Mobile Lipids + Lactate*	*1.33*
Alanine	1.47
*N-acetyl-aspartate + N-acetyl containing compounds + Mobile lipids*	*2.02*
*Glutamate + glutamine*	*2.10 − 2.40 + 3.80*
*PUFA (Mobile Lipids)*	*2.80*
GABA	3.00
*Total Creatine*	*3.03*
*Choline-containing compounds*	*3.21*
Scyllo-inositol	3.34
Taurine	3.42
*Myo-inositol + glycine*	*3.55*
Lactate	4.10

**Table 2:** Results of Ki67 percentage ± SD of fields corresponding to green and red pixels in nosological images, as well as the global percentage of Ki67 for the studied cases. Percentage of *TRI* and its classification by *TRI* criteria are also shown. n.a. = not available (the low number of green pixels observed in the case C979 (seven in Grid 1 and four in Grid 2), did not allow to confidently establish a field for corresponding histopathology evaluation in that tumor region).

Case	Ki67% ± SD (Green Fields)	Ki67% ± SD (Red Fields)	Ki67% ± SD (Global)	*TRI*%	Classification by *TRI* Criteria
C971	17.2 ± 16.8	25.7 ± 17.0	22.0 ± 17.2	44.1	Intermediate
C1022	19.0 ± 20.9	54.9 ± 32.6 *	42.5 ± 33.5	46.5	Intermediate
C1026	53.5 ± 30.3	73.0 ± 26.8	66.0 ± 39.1	38.9	Intermediate
C979	n.a.	64.8 ± 7.3	64.8 ± 7.3	3.3	Low
C1100	82.9 ± 4.6	92.5 ± 0.6	82.2 ± 7.7	66.3	High
C1108	75.1 ± 9.5	75.8 ± 0.2	79.3 ± 10.1	70.3	High
C1110	n.a.	63.9 ± 13.0	63.9 ± 13.0	0	Control
C1111	n.a.	73.3 ± 6.4	73.3 ± 6.4	0	Control

* = significant differences in Ki67% between red and green areas for this case.

## Supplementary Materials

The following are available online at www.mdpi.com/2218-1989/7/2/20/s1, Figure S1: Mean tumor volume evolution ± SD of different cases studied in this and previous work from the group, Figure S2: Mean ± SD evolution of body weight of mice studied by multi-slice MRSI, Figure S3: Graphical representation of the tumor volume evolution, and the percentage of responding “green” pixels obtained after NMF analysis of MRSI data acquired in the multi-slice set as in [13], Figure S4: Comparison of responding/non responding spectral patterns with expansions in some differential zones with tentative metabolite assignment as in Figure 5 and Table 1, Figure S5: Ki67 immunostainings in histological areas corresponding to red or green regions in nosological images, from different grids of chosen cases, Figure S6: Graph bars representing global Ki67% values, Figure S7: Boxplot of global number of Ki67 positive cells/mm2 of different groups of cases classified by *TRI* response, Figure S8: (A). Boxplot of volume of Ki67 positive cells in each *TRI* group; (B). Representation of percentage of acellular spaces observed in different TRI groups, Figure S9: TMZ administration schema with 3 therapy cycles with a 3-day interleave, as described previously by us [18], Table S1: Data summary of tumor volume the day before starting therapy administration and the day in which euthanasia was carried out, Table S2: Welfare parameters followed up in the study.

## Abbreviations

Ala	Alanine
Cho	Choline
Cre	Creatine
CTLs	Cytotoxic T-lymphocytes
DMPM	Dynamic MRSI processing Module
DWI	Diffusion Weighted Imaging
ETL	Echo train length
FOV	Field of view
GABRMN	*Grup d’Aplicacions Biomèdiques de la RMN*
GBM	Glioblastoma
GIC	Glioma-initiating cells
Glx	Glutamine + glutamate
HE	Hematoxylin-eosin
HR	High response
IQR	Interquartile range
Ins + Gly	Myo-inositol + glycine
IR	Intermediate response
Lac	Lactate
LR	Low response
ML	Mobile lipids
MM	Macromolecules
MTX	Matrix size
NA	Number of averages
NAA	*N*-acetyl-aspartate
NAc	*N*-acetyl group containing compounds
NMF	Non-negative matrix factorization
NS	Number of slices
PBS	Phosphate-buffered saline
p.i.	Post-inoculation
ppm	parts per million
PR	Pattern recognition
PUFA	Polyunsaturated fatty acids
RANO	Response assessment in neuro-oncology
RARE	Rapid acquisition with relaxation enhancement
ST	Slice thickness
TAT	Total acquisition time
TE_eff_	Effective echo time
TMZ	Temozolomide
TR	Repetition time
*TRI*	Tumor responding index
VOI	Volume of interest
3DiCSI	3D interactive chemical shift imaging
