# Supplementary material for: Metabolomics of Therapy Response in Preclinical Glioblastoma: A Multi-Slice MRSI-Based Volumetric Analysis for Noninvasive Assessment of Temozolomide Treatment
Source: Metabolites. 2017 May 18;7(2):20. doi: 10.3390/metabo7020020 (PMC5487991; doi:10.3390/metabo7020020)
Supplement: Supplementary file 1 [file metabolites-07-00020-s001.pdf]

Supplementary Materials: Metabolomics of Therapy Response in Preclinical Glioblastoma: A Multi-slice MRSI-Based Volumetric Analysis for Noninvasive Assessment of Temozolomide Treatment

Nuria Arias-Ramos, Laura Ferrer-Font, Silvia Lope-Piedrafita, Victor Mocioiu, Margarida Julià-Sapé, Martí Pumarola, Carles Arús and Ana Paula Candiota

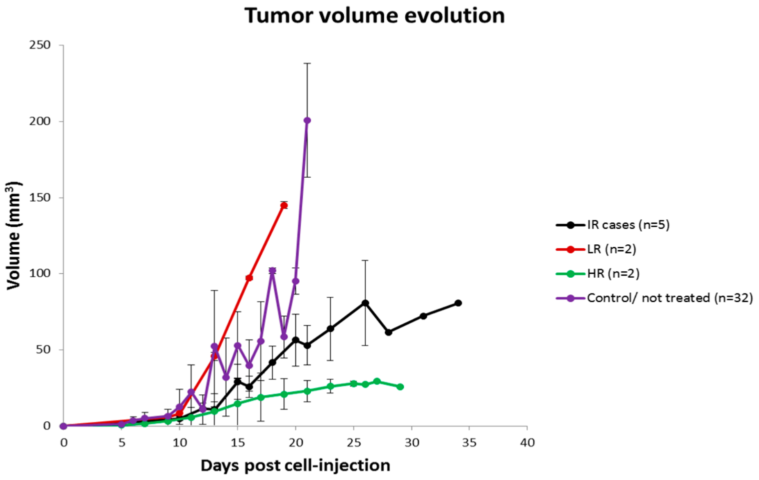

Figure S1. Mean tumor volume evolution ± SD of different cases studied in this and previous work from the group. IR cases until euthanization day (*n* = 5, case C974 was not included because it was followed up until endpoint) are represented with black line; LR cases (*n* = 2) with red line; HR cases (*n* = 2) with green line and control cases (*n* = 32, in addition to the 4 control cases explored in this work, 28 additional cases from previous work from our group were added to confirm consistent evolution pattern by MRI follow-up) with purple line. Significant differences ($p < 0.05$) were observed for tumor volume evolution between IR cases and HR cases; and between LR cases and control cases. The TMZ administration period is not represented in the graph due to different schedules used in HR cases. See supplementary table 1 for euthanization days in each case. This graph is not representative of the overall survival of mice because mice were euthanized at specific time points to perform histopathological validation (i.e., it was not an endpoint study).

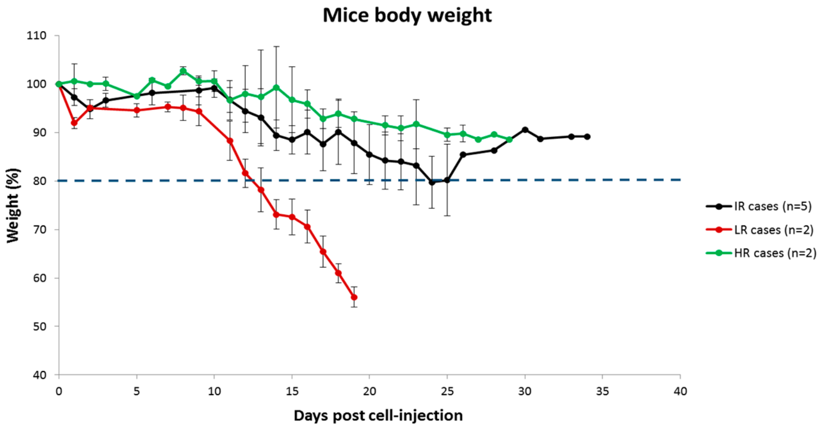

Figure S2. Mean ± SD evolution of body weight of mice studied by multi-slice MRSI (mouse weight at day of cell injection (day 0 p.i.) was considered 100%). IR cases are represented in black (C974 is not included in this graph because it was followed up until endpoint, and at that point it could be no longer classified as an IR case), LR cases are represented in red and HR cases are represented in green. The dashed blue line indicates the 20% weight reduction point. Significant differences ($p < 0.05$) in body weight evolution were observed between all groups.

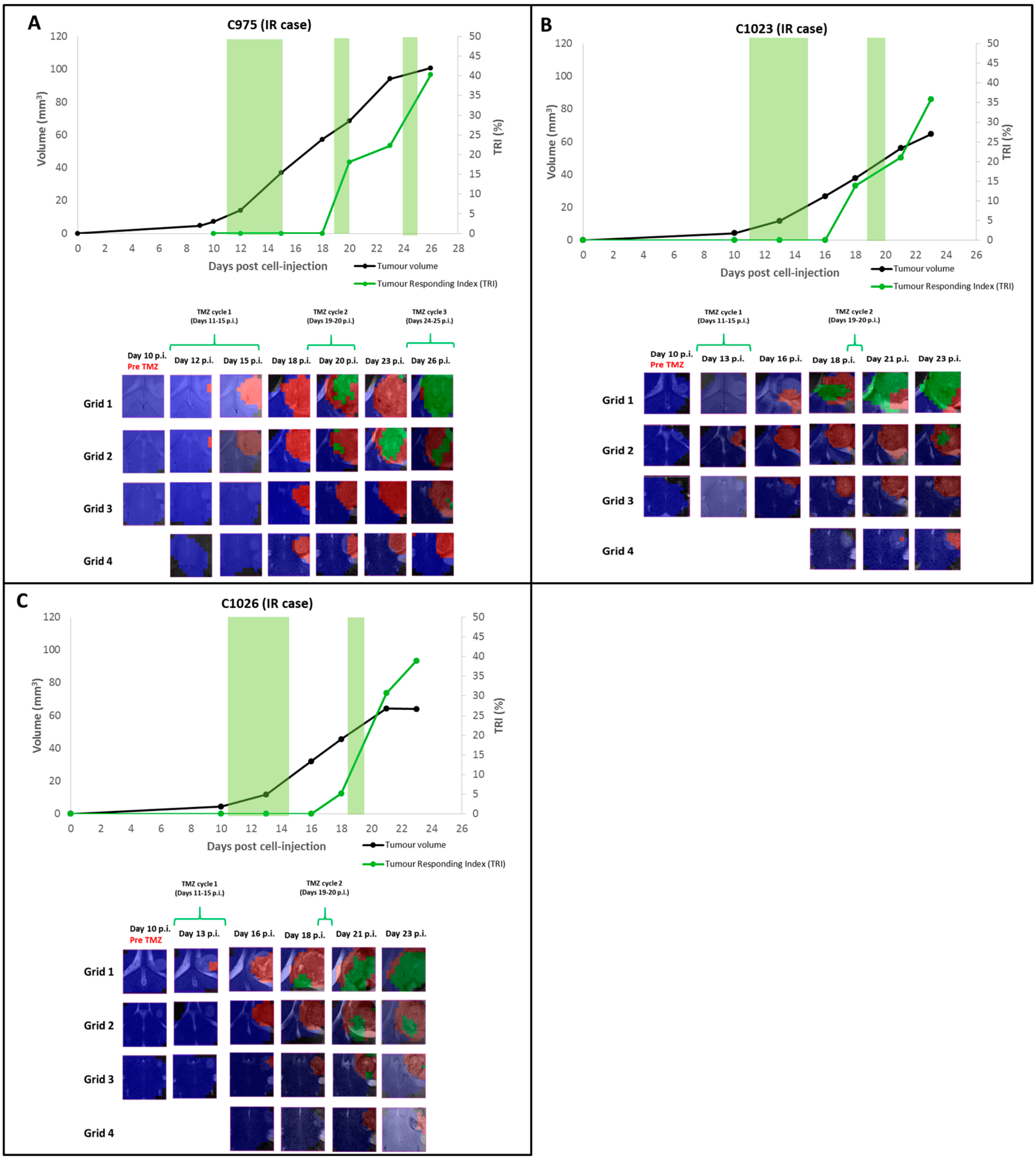

Figure S3. Graphical representation of the tumor volume evolution (in mm^3, *black* line, left axis), and the percentage of responding "green" pixels obtained after NMF analysis of MRSI data acquired in the multi-slice set as in [1] (in %, green line, right axis). For chosen time points, the evolution of the nosological images obtained with the semisupervised source extraction system is shown in four columns of colour coded grids, superimposed to the T_{2w}-MRI for each slice. The green shaded columns indicate TMZ administration periods. (**A**). Corresponds to the IR case C975, green pixels were observed first in grid 1 and a few in grid 2 at day 20 p.i. At day 23 p.i., green pixels were observed only in grid 2 and at day 26 p.i., green pixels were observed in grids 1 and 2 with a *TRI* = 40.3%. Tumor volume variation meeting criteria for 'stable disease' according to RECIST was observed from day 23 to 26 p.i. (**B**). Corresponds to the IR case C1023, green pixels were observed in grid 1 at days 18 and 21 p.i., at day 23 p.i. green pixels were observed in grids 1 and 2 (*TRI* = 35.8%), this TRI increase coincides with the possible beginning of a stable disease stage (volume increase with respect to preceding observation of 15.1%). (**C**). Corresponds to the IR case C1026, with an evolution similar to the C1023 case, green pixels were observed only in grid 1 at day 18 p.i. while green pixels were observed in grids 1 and 2 at days 21 and 23 p.i. (*TRI* = 38.9%, at day 23 p.i.). From day 21 to 23 p.i., the beginning of a stable disease stage was also observed.

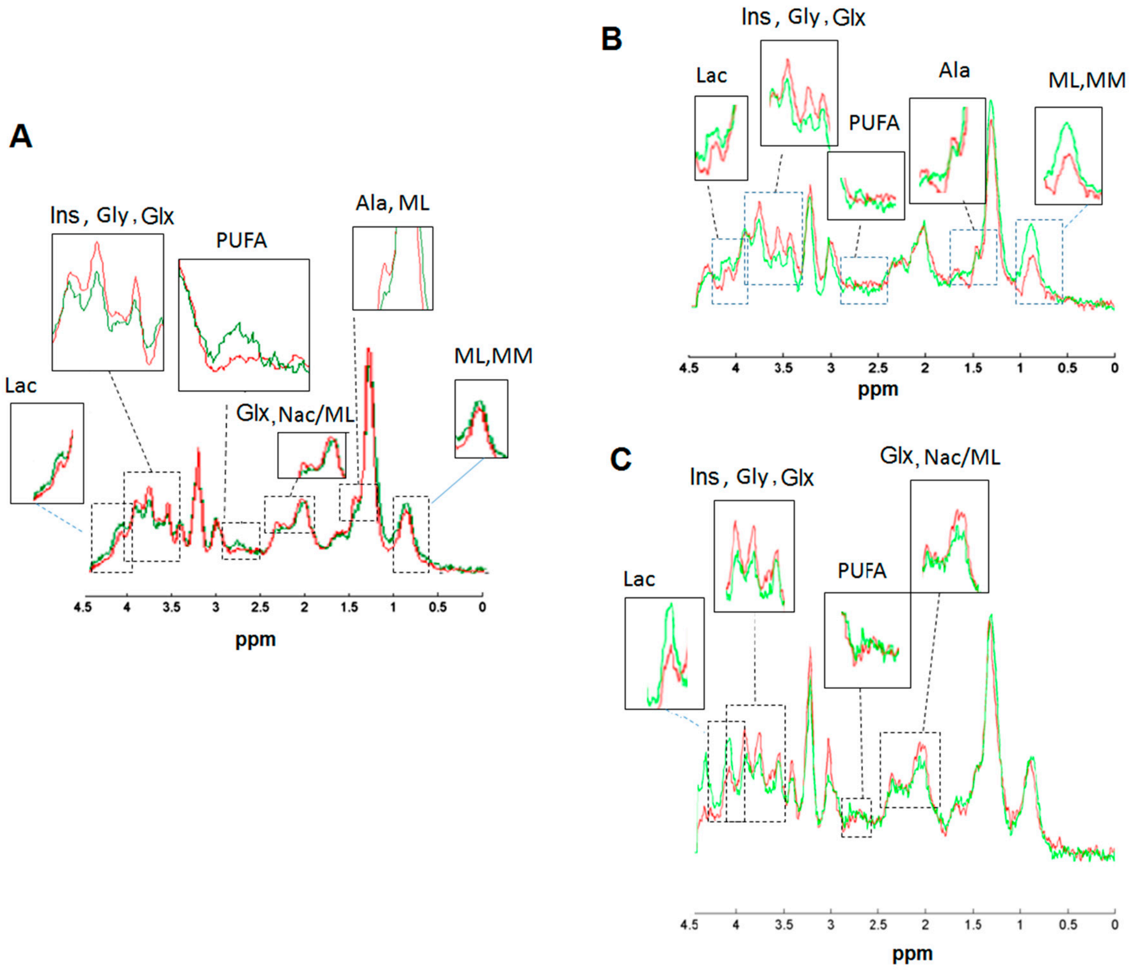

Figure S4. Comparison of responding/non responding spectral patterns with expansions in some differential zones with tentative metabolite assignment as in Figure 5 and Table 1. (**A**) Prototypical patterns (sources) extracted from TMZ treated (green) and untreated (read) tumor-bearing mice as described in [1]. (**B**) Superimposition of average spectra from an untreated case (n = 44, red voxels, C1112 day 13 p.i., grid 2) and a treated case (n = 32, green voxels, C1108 day 29 p.i., grid 1). (**C**) Superimposition of average spectra from the zones classified either as non-responding (n = 35, red) or responding (n=26) from mouse C971, an IR case. ML, MM: mobile lipids + macromolecules; Ala, ML: alanine + mobile lipids; NAc/ML: N-acetyl containing compounds + mobile lipids; PUFA: polyunsaturated fatty acids in mobile lipids; Glx, glutamine + glutamate; Gly: glycine (glycine signal is also contributed by myo-inositol, Ins); Lac: lactate. Tentative resonance assignment according to [2–13].

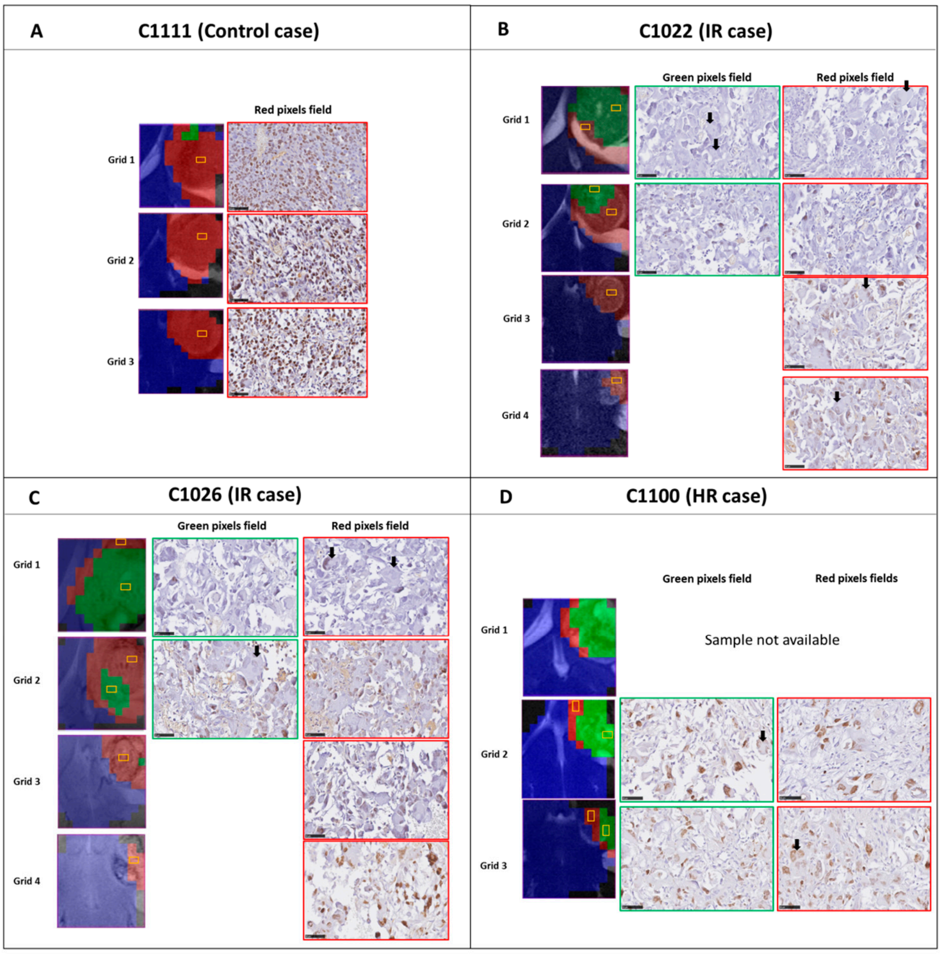

Figure S5. Ki67 immunostainings (40× magnification, the scale bar corresponds to 50 μm) in histological areas corresponding to red or green regions in nosological images, from different grids of chosen cases. The nuclei of Ki67 positive cells are stained in brown. (**A**). Control case C1111, with a global Ki67 of 73.3 ± 6.4% and a typical tumoral morphology, with small to medium-sized polygonal or irregular cells with rounded nuclei and scanty cytoplasm without any giant cells. (**B**). IR case C1022, presented lower Ki67 immunostaining (42.5 ± 33.5%) than control and LR cases, with presence of giant cells with several nuclei (black arrows), and acellular spaces. (**C**). IR case C1026, presented as well as in case C1022 with lower Ki67 immunostaining (66.0 ± 39.1%) than control and LR cases, and presence of giant cells (black arrows), and acellular spaces. (**D**). HR case C1100, with a higher value of Ki67 (82.2 ± 7.7%) compared to control, LR and IR cases, although large acellular spaces among cells and giant cells (black arrows) were observed. In this case, no histopathological evaluation could be performed from the first grid.

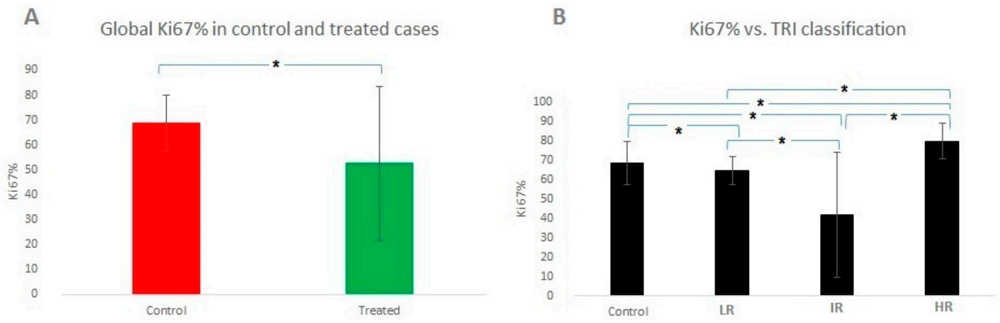

Figure S6. Graph bars representing global Ki67% values (mean ± SD). (**A**). Global Ki67% was higher in control cases than in treated cases. (**B**). Ki67% in different cases classified by TRI criteria. A descending trend was observed between control, LR and IR cases, as expected. An unexpectedly high Ki67% value was found in HR cases. *: $p < 0.05$ with Mann–Whitney's U test.

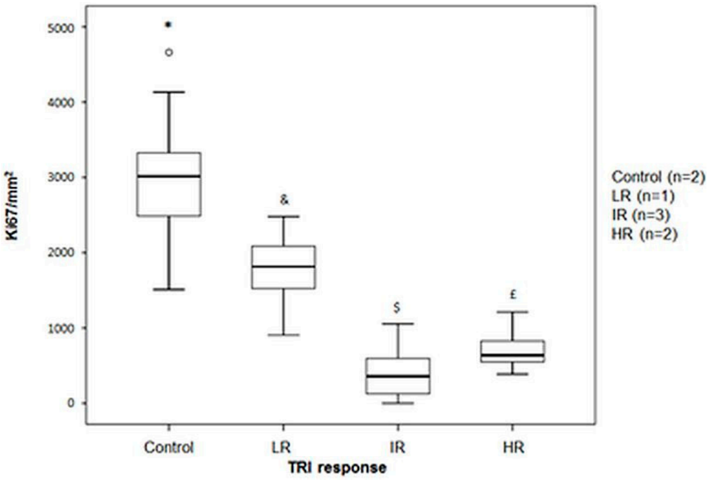

Figure S7. Boxplot of global number of Ki67 positive cells/mm² of different groups of cases classified by TRI response. Boxplot features are the same as explained in Figure 10. *: $p < 0.05$ in comparison with LR, IR and HR cases; &: $p < 0.05$ in comparison with control, IR and HR cases; $: $p < 0.05$ in comparison with control, LR and HR cases; £: p < 0-05 in comparison with control, LR and IR cases.

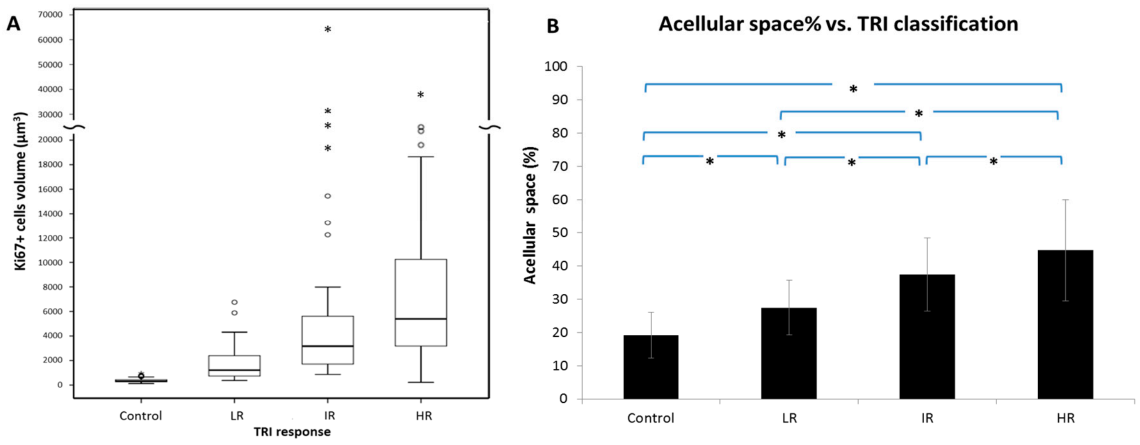

Figure S8. A. Boxplot of volume of Ki67 positive cells in each TRI group (cells were analyzed in one representative field of each of the following cases: 2 HR, 1 LR, 3 IR and 2 control, and the volumes found were represented in boxplot format). Ki67 positive cells showed a smaller volume in control than in treated cases; and in treated cases, bigger cells were observed as increase in TRI response was observed. B. Representation of percentage of acellular spaces observed in different TRI groups: an increase in such acellular spaces was observed in treated cases, the more TRI response classification, the more acellular spaces were observed. Boxplot features are the same as explained in Figure 9. *: $p < 0.05$ with Mann–Whitney's U test.

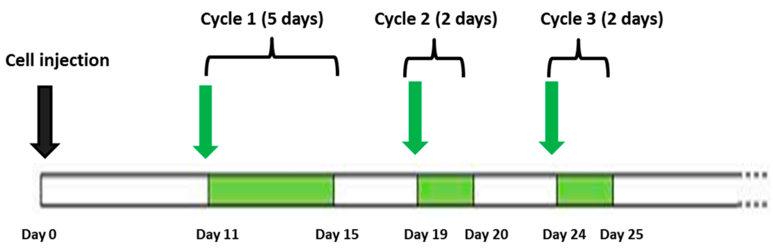

Figure S9. TMZ administration schema with 3 therapy cycles with a 3-day interleave, as described previously by us [10]. The starting day of each cycle is marked with green arrows. The duration of each cycle (5 days for the first cycle, 2 days for second and third cycle) was the same for mice groups A and B. The cycle shown in this figure is the corresponding to group A. For group B, the starting day was adapted to tumor volume (see section 2.2 of the main manuscript).

Table S1. Data summary of tumor volume the day before starting therapy administration and the day in which euthanasia was carried out. Classification with RECIST and TRI criteria the day of euthanasia.

Case	Tumor Volume (mm^3) (pre therapy)	Tumor Volume (mm^3) (day of euthanasia)	Classification by RECIST Criteria	Classification by TRI Criteria
C971	5.0	80.7 (34 p.i.)	Stable disease	Intermediate
C974	7.3	220.5 (40 p.i.)	Stable disease	Intermediate
C975	7.2	100.7 (26 p.i.)	Stable disease	Intermediate
C1022	3.1	35.7 (23 p.i.)	Stable disease	Intermediate
C1023	4.3	64.7 (23 p.i.)	Stable disease	Intermediate
C1026	4.4	64.0 (23 p.i.)	Stable disease	Intermediate
C979	8.4	146.7 (19 p.i.)	Progressive disease	Low
C981	8.1	143.5 (19 p.i.)	Progressive disease	Low
C1100	2.9	27.3 (26 p.i.)	Stable disease	High
C1108	3.0	25.7 (29 p.i.)	Stable disease	High
C1109	Control	47.5 (11 p.i.)	Progressive disease	Control
C1110	Control	81.7 (13 p.i.)	Progressive disease	Control
C1111	Control	88.9 (16 p.i.)	Progressive disease	Control
C1112	Control	88.7 (13 p.i.)	Progressive disease	Control

-Although mean values of tumor volumes pre-therapy were different between groups (HR 2.95±0.07 mm^3, IR 5.21±1.69 mm^3, LR 8.25±0.21mm^3), there were not enough cases to perform statistical tests. The same was observed for tumor volume at the euthanization day (HR 26.50±1.13 mm^3, IR 94.38±65.39 mm^3, LR 145.1±2.26 mm^3)

-Control mice were euthanized when tumor volume was between 50–80 mm^3 ± 10%, in order to avoid reaching necrotic stage in tumors, when higher heterogeneity in MRI and MRSI would be expected, which could complicate the interpretation of the results.

Table S2. Welfare parameters followed up in the study.

Parameter for Endpoint	Means of Verification
Weight loss above 20% regarding the previous register	Scale readings
Marked piloerection	Piloerection detected during animal observation
Animal shows subdued behaviour patterns even when provoked	Apathic behaviour during weighting procedure, in comparison with control animals.
Intermittent or persistent tremors	Observation of animals before and after weighting procedure

References

1. Delgado-Goñi, T.; Ortega-Martorell, S.; Ciezka, M.; Olier, I.; Candiota, A. P.; Julià-Sapé, M.; Fernández, F.; Pumarola, M.; Lisboa, P. J.; Arús, C. MRSI-based molecular imaging of therapy response to temozolomide in preclinical glioblastoma using source analysis. *NMR Biomed.* **2016**, *29*, 732–743.
2. Remy, C.; Arus, C.; Ziegler, A.; Lai, E. S.; Moreno, A.; Le Fur, Y.; Decorps, M. In vivo, ex vivo, and in vitro one- and two-dimensional nuclear magnetic resonance spectroscopy of an intracerebral glioma in rat brain: assignment of resonances. *J. Neurochem.* **1994**, *62*, 166–179.
3. Barba, I.; Cabanas, M. E.; Arus, C. The relationship between nuclear magnetic resonance-visible lipids, lipid droplets, and cell proliferation in cultured C6 cells. *Cancer Res.* **1999**, *59*, 1861–1868.
4. Barba, I.; Mann, P.; Cabanas, M. E.; Arus, C.; Gasparovic, C. Mobile lipid production after confluence and pH stress in perfused C6 cells. *NMR Biomed.* **2001**, *14*, 33–40.
5. Valverde, D.; Quintero, M. R.; Candiota, A. P.; Badiella, L.; Cabanas, M. E.; Arus, C.; Cabañas, M. E.; Arús, C. Analysis of the changes in the 1H NMR spectral pattern of perchloric acid extracts of C6 cells with growth. *NMR Biomed.* **2006**, *19*, 223–230.
6. Simões, R. V; García-Martín, M. L.; Cerdán, S.; Arús, C. Perturbation of mouse glioma MRS pattern by induced acute hyperglycemia. *NMR Biomed* **2008**, *21*, 251–264.

7. Simoes, R. V; Delgado-Goñi, T.; Lope-Piedrafita, S.; Arus, C. 1H-MRSI pattern perturbation in a mouse glioma: The effects of acute hyperglycemia and moderate hypothermia. *NMR Biomed.* **2010**, *23*, 23–33.
8. Davila, M.; Candiota, A. P.; Pumarola, M.; Arus, C. Minimization of spectral pattern changes during HRMAS experiments at 37 degrees celsius by prior focused microwave irradiation. *Magn Reson Mater Phy.* **2012**, *25*, 401–410.
9. Candiota, A. P.; Majos, C.; Bassols, A.; Cabanas, M. E.; Acebes, J. J.; Quintero, M. R.; Arus, C. Assignment of the 2.03 ppm resonance in in vivo 1H MRS of human brain tumour cystic fluid: contribution of macromolecules. *Magn Reson Mater Phy.* **2004**, *17*, 36–46.
10. Delgado-Goñi, T.; Julià-Sapé, M.; Candiota, A. P.; Pumarola, M.; Arús, C. Molecular imaging coupled to pattern recognition distinguishes response to temozolomide in preclinical glioblastoma. *NMR Biomed.* **2014**, *27*, 1333–1345.
11. Hakumäki, J. M.; Poptani, H.; Sandmair, A. M.; Ylä-Herttuala, S.; Kauppinen, R. A. 1H MRS detects polyunsaturated fatty acid accumulation during gene therapy of glioma: implications for the in vivo detection of apoptosis. *Nat. Med.* **1999**, *5*, 1323–1327.
12. Govindaraju, V.; Young, K.; Maudsley, A. A. Proton NMR chemical shifts and coupling constants for brain metabolites. *NMR Biomed.* **2000**, *13*, 129–153.
13. Hulsey, K. M.; Mashimo, T.; Banerjee, A.; Soesbe, T. C.; Spence, J. S.; Vemireddy, V.; Maher, E. A.; Bachoo, R. M.; Choi, C. (1)H MRS characterization of neurochemical profiles in orthotopic mouse models of human brain tumors. *NMR Biomed.* **2015**, *28*, 108–115.
